# Coupled Ecosystem/Supply Chain Modelling of Fish Products from Sea to Shelf: The Peruvian *Anchoveta* Case

**DOI:** 10.1371/journal.pone.0102057

**Published:** 2014-07-08

**Authors:** Angel Avadí, Pierre Fréon, Jorge Tam

**Affiliations:** 1 Université Montpellier 2– Sciences et Techniques, Montpellier, France; 2 Institut de Recherche pour le Développement (IRD), UMR212 EME IFREMER/IRD/UM2, Sète, France; 3 Instituto del Mar del Perú (IMARPE), Callao, Peru; Institute of Marine Research, Norway

## Abstract

Sustainability assessment of food supply chains is relevant for global sustainable development. A framework is proposed for analysing fishfood (fish products for direct human consumption) supply chains with local or international scopes. It combines a material flow model (including an ecosystem dimension) of the supply chains, calculation of sustainability indicators (environmental, socio-economic, nutritional), and finally multi-criteria comparison of alternative supply chains (e.g. fates of landed fish) and future exploitation scenarios. The Peruvian *anchoveta* fishery is the starting point for various local and global supply chains, especially via reduction of *anchoveta* into fishmeal and oil, used worldwide as a key input in livestock and fish feeds. The Peruvian *anchoveta* supply chains are described, and the proposed methodology is used to model them. Three scenarios were explored: *status quo* of fish exploitation (Scenario 1), increase in *anchoveta* landings for food (Scenario 2), and radical decrease in total *anchoveta* landings to allow other fish stocks to prosper (Scenario 3). It was found that Scenario 2 provided the best balance of sustainability improvements among the three scenarios, but further refinement of the assessment is recommended. In the long term, the best opportunities for improving the environmental and socio-economic performance of Peruvian fisheries are related to sustainability-improving management and policy changes affecting the reduction industry. Our approach provides the tools and quantitative results to identify these best improvement opportunities.

## Introduction

Sustainability in food systems has several dimensions of concern, including environmental [1], [2], socio-economic and food security [3], [4], consumption patterns [5], technology [6], information [7] and governance/policy [8]. Moreover, sustainability arises from the complex interrelation among these factors, and thus science should focus on the most significant cause-and-effect relationships and driving forces that shape these interrelations so as to inform and provide tools for management and policy [9].

A recent journal editorial stressed the growing challenges of sustainability in food systems, given the increasing demand for food and the environmental impacts associated with modern food production [10]. The editorial referred to the relevance of trade policy and trade impacts on vulnerable communities, as well as to the need for globally-accepted metrics and policies for sustainability. This kind of narrative is representative of the general interest of the research community in studying and advancing sustainability tools for policy and decision-making. Agricultural and fishfood systems feed the world. We use the term “fishfood” to describe edible products from marine and freshwater fisheries and aquaculture. Despite the relatively small size of the global fishfood economic system in comparison to agriculture, it encompasses complex socio-economic networks with considerable impact of the world’s environment. Economically, fishfood products represent about 10% of the value of total agricultural exports, and this percentage is increasing. Nutritionally, fish represent over 20% of animal protein intake in low-income and food-deficient countries [11], [12]. Therefore, it is imperative to apply sustainability principles to the design, operation and assessment of fishfood systems.

“Fishfood system” is used here an umbrella term for complex systems producing fish directly consumed by humans, and closely interacting with surrounding aquatic and terrestrial ecosystems. Resource management science and research have produced a variety of approaches for capturing interactions between natural and socio-economic realms in such systems.

An essential feature of all approaches for understanding complex systems is modelling [13]. The ideal level/zone of complexity of modelling has been defined as the level of resolution at which essential real-world dynamics are included and analysis is not too burdensome [14] [15].

In fisheries, ecological processes such as predation, competition, environmental regime shifts, and habitat effects have the potential to impact bio-economic dynamics (e.g. recovery of exploited stocks, surplus production) [16]. These impacts may manifest themselves at an order of magnitude comparable to that exerted by fisheries pressure. Ecological/ecosystem modelling is a rich, well established research field; nonetheless, it is not always included in fisheries modelling and management [16]. Whole ecosystem models try to account for all trophic levels in the ecosystems studied. Some of the most notable examples are ECOPATH [17] and ECOSIM [18]. Currently, the most commonly used whole ecosystem modelling approach is probably Ecopath with Ecosim (EwE), a combination of ECOPATH, ECOSIM and a constantly increasing number of add-ons [19]. A software implementation of EwE is freely available for evaluating ecosystem impacts of fisheries [20], [21]. EwE modelling is data-intensive, especially regarding biomasses and diets, and its outputs require interpretation to be used for policy-making support, among other limitations [21].

The “supply chain” is a concept used since the early 1980s to refer to dynamics between firms (value chains) contributing to the provision of a good or service. It encompasses all value chains, integrated or not, along the life cycle of the delivered product [22], as well as material, information and financial flows circulating among these value chains [23]. The supply chain concept is the ideal approach for studying today’s economic organisations, immersed in a globalised world and both featuring and lacking vertical integration. Supply chain modelling is performed to understand, analyse and improve the efficiency, effectiveness and sustainability of supply chains. Supply chain modelling theory has been extensively applied to the study of food supply chains. Goals of supply chain modelling in food systems include cost reduction, safety, quality, flexibility and responsiveness, among other aspects [24]. Supply and value chain analysis, as well as modelling approaches, have been applied to fisheries, aquaculture and whole fishfood supply chains, as extensively reviewed in [25]. Non-modelling studies have focused on reducing costs, increasing efficiency and improving product quality, as well as (more recently) in developing or re-shaping existing supply chains [26].

Models oriented toward operations research have diverse objectives, depending on the system under study. In fisheries, aspects such as resource allocation problems, uncertainty management, harvest policy and strategy, harvest timing, quota decisions, experimental management regimes, investment in fleet capacity, and stock switching by fishermen [25] are studied. In aquaculture, trade-offs among alternative activities, strategic planning requirements for emerging technologies, planning and management, optimal harvesting time and other optimal control frameworks, feeding regimes, and risk management are studied [25]. Modelling of whole fishfood supply chains is less common; thus, it has been suggested that future research should focus on optimal production planning, costs associated with additional sorting of raw materials (due to the batch nature of many landed species) and quality aspects [24]. Past research has focused on handling and preservation practices for extended shelf life [26].

Although supply chain analysis and modelling of agrifood systems is quite common in research, modelling of fishfood supply chains is less common. Few models combine ecosystems and (fishfood) supply chains. The few social-ecological systems models applied to fisheries (as listed in [13]) and fisheries bio-economic models (e.g. those listed in [27], [28]) are spatially explicit and include fishermen/vessel behaviour and their impact on management systems. Despite these few examples, most fisheries-related modelling research has historically focused on ecological (or ecosystem) modelling, that is to say, on ecosystem-fisheries interactions which do not explore socio-economic aspects.

Combining a fish supply-chain modelling approach with an EwE trophic model to model policy scenarios for stock recovery was first proposed in [29]. This approach was based on an idea later published in [30], in which a social-ecological system model combining ecosystem (using EwE trophic models) and proprietary value-chain modelling approaches was proposed. The model coupling (partial two-way interactions limited to the feedback effect of the producers on the ecosystem) proposed in [30] was eventually implemented as a plug-in for EwE 6.2. The coupled model was recently used in a case study [31]. We borrowed the one-way vs. two-way coupling wording and criteria from ecosystem modelling and used it to define the types of interactions between an ecosystem model and a material flow (supply chain) model (MFM). More details on the classification of modelling tools and justification of the models retained is presented in section A in File S1.

In this article, a sustainability modelling and assessment methodology is proposed and applied to compare several fishfood and agricultural supply chains that compete for Peruvian *anchoveta* (*Engraulis ringens*) resources. These chains generate a variety of impacts on Peruvian ecosystems and society, as well as on the global environment and economy. Therefore, we compare relative environmental and socio-economic performance of products from the chains and analyse alternative exploitation and fish fate (final fishfood product) scenarios. Ultimately, we track the fate of one t of landed *anchoveta* channelled through alternative Peruvian supply chains, now and in the future. The system under study encompasses the supply chains from extraction (fisheries and their impact on the Northern Humboldt Current ecosystem), through reduction activities for fishmeal and fish oil, aquafeed production (taking into account other agricultural inputs to aquafeeds), aquaculture, fishfood industries and, finally, to fishfood products on grocery shelves.

The dynamics of these complex supply chains have never been studied in a holistic, sustainability-imbued way. Understanding these dynamics and impacts to the largest extent possible is the motivation of this research, so that decision makers along the chains are informed and actions are taken to improve sustainability of the *anchoveta*-based fishfood fisheries and industries.

The research topic connects with the wider topic of sustainability assessment of food systems, and its importance derives from the prevalence of Peruvian fishmeal in international food supply chains, as Peru is by far the largest global exporter of fishmeal and fish oil used to supply aquaculture and animal production supply chains, mainly in Asia and Europe [12]. Simultaneously, since Peru is a developing country facing nutritional and social challenges, the fact that most fisheries landings are destined for reduction into fishmeal and fish oil is subject to discussion and multi-disciplinary analysis [31], [32].

This article first introduces the Peruvian *anchoveta* supply chains and the sustainability assessment framework. It then presents the results obtained from applying the framework to the Peruvian case study by assessing and comparing the sustainability of supply chains and alternative exploitation scenarios. Finally, the methodology is discussed in relation to the results obtained, and suggestions for improving both the current and (possible) future situations are proposed.

## Peruvian *Anchoveta* Supply Chains

### The Humboldt Current System

The Northern Humboldt Current System (NHCS) identifies the tropical ocean area off Peru and north of Chile. The NHCS is considered the most productive fishing ground in the world because it produces more fish per area than any other region. Moreover, the NHCS has several unique characteristics that determine its productivity [33]. The NHCS is an eastern boundary upwelling ecosystem, extremely sensitive to climatic dynamics. Temperature anomalies, mainly associated with El Niño-Southern Oscillation (ENSO) and Pacific Ocean regime shifts, have historically produced huge changes in seabird populations and fluctuations in abundance of two numerically dominant species of pelagic fish: *anchoveta* and sardine (*Sardinops sagax*). *Anchoveta* is one of the world’s largest exploited fish stocks.

### The *anchoveta* fishery

The modern *anchoveta* fishery started in Peru around 1955, parallel with the decline of the previously profitable guano industry. The 1957–58 ENSO event decimated guano-producing seabird populations and coincided with further development of the *anchoveta* fishery. During the 1960s the fleet and the fishery grew continuously until 1970, peaking with the largest historical harvest of 12.3 million t, representing 20% of that year’s world catch of all fish [33]. In 1972, the *anchoveta* stock collapsed, probably due to combination of high fishing pressure, a regime shift in the ecosystem and a strong ENSO event, followed by a slow recovery of the *anchoveta* stock and catches as well as changes in fisheries management and legislation [34] (Figure B1 in File S1). From 2000 to 2009, catches were stable compared to historical landings, averaging 7.1 million t per year. In 2010, an ENSO event and management measures reduced landings to 3.4 million t [12], [35].

Currently, Peruvian fisheries are ruled by the currently valid Fisheries Act (Decree Law 25977 of 1992) and its applicable by-laws (Supreme Decree 012-2001-PRODUCE, Supreme Decree 005-2012-PRODUCE). The Peruvian purse-seiner fishery is the world’s largest mono-specific fishery, both in landings and in number of vessels [33], [36], [37]. The fleet is heterogeneous. The industrial fleet (vessels with holding capacity >32.6 m^3^) includes steel vessels and wooden vessels nicknamed “Vikingas”. As of 2012, ∼660 industrial steel vessels (operating directly under regime Decree Law 25977) target *anchoveta* for reduction (i.e. for fishmeal plants). Additionally, almost 700 Vikingas (operating under regime Law No. 26920) also target *anchoveta* for reduction. The small-scale fleet includes vessels with holding capacity <10 m^3^, while the medium-scale fleet has vessels with holding capacity of 10–32.6 m^3^. Small-scale vessels also differ from medium-scale ones in the level of technology and capture systems used; small-scale vessels are characterised by manual labour and basic technology [38]. In total, the small- and medium-scale (SMS) fleet includes about 850 wooden vessels that by law target *anchoveta* (among other species) only for direct human consumption (DHC), but also illegally for reduction fishmeal plants. As a result, a small percentage of national catches is rendered into seafood products for DHC, according to both official PRODUCE statistics and IMARPE comprehensive data [34] as detailed in [39], [40]. PRODUCE is the Peruvian Ministry of Production (www.produce.gob.pe/), while IMARPE is the Peruvian Marine Research Institute (Instituto del Mar del Perú, www.imarpe.pe/imarpe/), a public institution leading national research on marine resources and the marine environment.

Catches by the steel fleet represent around 81% of the total *anchoveta* catches for reduction, while the Vikingas capture 19%, according to IMARPE statistics (Marilú Bouchon, unpublished data). The industrial fleet landings for indirect human consumption (IHC) (i.e. reduction) represent >99% of total catches, while SMS fleet landings for DHC (fresh, freezing, canning, curing) represent <1% of total catches, according to PRODUCE statistics, as summarised in Table 1.

**Table 1:** Statistics for *anchoveta* landings and processing (2001–2011).

Year	2001	2002	2003	2004	2005	2006	2007	2008	2009	2010	2011	Average
*Anchoveta* landings	6 358 217	8 104 729	5 347 187	8 808 494	8 655,461	5 935 302	6 159 802	6 257 981	5 935 165	3 450 609	7 103 061	**6 556 001**
*Anchoveta* for reduction	6 347 600	8 082 897	5 335 500	8 797 100	8 628 400	5 891 800	6 084 700	6 159 387	5 828 600	3 330 400	6 994 051	**6 498 221**
Fishmeal production^a^	2 034 900	1 562 116	1 416 500	1 807 000	2 067 900	1 367 900	1 284 500	1 585 600	1 584 100	1 119 300	1 235 674	**1 551 408**
National consumption	91 800	46 686	43 700	53 600	66 400	25 400	20 700	20 800	36 700	33 600	-	**39 944**
Exports	1 943 100	1 515 430	1 372 800	1 753 400	2 001 500	1 342 500	1 263 800	1 564 800	1 547 400	1 085 700	-	**1 399 130**
Fish oil production	447 200	206 150	267 508	363 000	339 400	346 773	371 600	280 400	335 000	320 800	248 637	**320 588**
National consumption	131 800	45 245	80 800	78 200	60 600	58 200	65 900	41 800	46 800	69 700	-	**61 731**
Exports	315 400	160 905	186 708	284 800	278 800	288 573	305 700	238 600	288 200	251 100	-	**236 253**
*Anchoveta* for DHC	10 617	21 832	11 687	11 394	27 061	43 502	75 102	98 594	106 565	120 209	109 010	**57 779**
Canning	3 286	13 364	4 823	2 631	14 887	31 000	61 944	78 851	84 957	94 234	84 194	**43 106**
Freezing	1 137	4 326	655	214	1 405	1 268	5 286	12 265	11 517	15 160	14 680	**6 174**
Fresh fish	398	9	392	320	348	538	401	336	293	223	44	**300**
Curing	3 717	4 132	5 806	8 194	10 425	10 658	7 459	7 142	9 762	10 579	10 092	**7 997**

a all species, >90% *anchoveta*. Based on PRODUCE data [48], [117], [121]. DCH: Direct Human Consumption.

Overcapitalisation/overcapacity affects the *anchoveta* fleets, largely due to the existence of a semi-regulated open access system that existed until the 2008 fishing season (inclusive) and a single national quota (Total Allowable Catch, TAC) that is revised each fishing season. Overcapitalisation is still substantial in Peru; in 2007 the fishing fleet was estimated to be 2.5–4.6 times its optimal size [41].

The Peruvian *anchoveta* fishery operates in two well-defined coastal areas in the South Pacific, as determined by the species habitat and behaviour: the north-central area (from 4°–14° S) and the south area (from 15° to ∼18° S, which continues in Chile from parallels ∼18° to 24° S). More detailed descriptions of the industrial steel, semi-industrial and SMS fleets are presented in [42], while discussions on their environmental performance are presented in [39].

### The reduction industries

Fishmeal plants produce fishmeal as the main product and fish oil as co-product. Inclusion of fishmeal and fish oil in aquafeeds has decreased [43] as alternative protein sources have become available and their effectiveness has been demonstrated. Nonetheless, the demand for fish reduction products has remained constant due to expansion of aquaculture, which consumed 61% of all fishmeal and 74% of all fish oil produced in 2008, and to continuous growth of livestock feed and pet food industries [12], [43], [44]. Peruvian fishmeal and fish oil represented 40–47% and 34–47% of the world’s supply from 2007–2011, respectively [45].

In Peru, more than 98% of fishmeal produced is derived from *anchoveta*. Plants can be classified into conventional, high-protein and residual, according to the technology used and product quality obtained ([46], [47]). Peruvian product labels describe “fair average quality” fishmeal (∼64% protein), dried with direct heat, “high protein content” fishmeal (67–70% protein), dried with indirect heat (steam, hot air), and residual fishmeal (processing residues, ≤55% protein), dried with direct heat.

Peru had 160 industrial fishmeal plants in 2012, but not a single registered artisanal fishmeal plant, according to [48]. Fifty percent of plants are concentrated in the northern coastal region, mainly in Chimbote and Chicama [49].

The reduction industry suffers from overcapacity: in 2007 the industry was 3–9 times its optimal size [41]. Although the 1992 General Fisheries Act prohibited further increase in capacity of reduction plants, the overcapacity issue was worsened by privatisation of the sector in the 1990s and many mergers and acquisitions from 2006–2008 that concentrated the sector [41]. A shift towards better technology, and thus better and more lucrative products, is noticeable in the increase in high-protein fishmeal processing capacity and production (fair average quality fishmeal from 37.6% in 2010 to 34.0% in 2011; prime fishmeal from 62.4% in 2010 to 66.0% in 2011) [50], [51].

Production and export of fishmeal and fish oil is the main driver for the thriving *anchoveta* industry. Peruvian fishmeal and oil are exported, among other aquaculture-producing countries, to China, Chile and some European countries. The main users of these imports are farms producing shrimp, salmonids, carp, tilapia and other cultivated species. It has been suggested that Chinese carp cultures may be the largest single consumer of fishmeal, despite low inclusion rates in feeds, due to the enormous volume of production [12], [52]. Other authors suggest shrimp farming in China as the main consumer (Patrik Henriksson, SEAT, pers. comm., 2012).

The fish-to-fishmeal conversion ratio in the Peruvian industry has increased from more than 5∶1 in the early 1990s to ∼4.2∶1 in recent years. Conversion ratios below 4.2 are considered impossible in the Peruvian context [41]. Table 2 compares several reported conversion ratios. Fish oil conversion ratios fluctuate greatly because they depend on the lipid content of *anchoveta*, which varies over time. The mean yield from 2001–2011 was 21.3∶1, as calculated based on statistics from PRODUCE and [48]. A more detailed discussion about the reduction industry is under preparation by our team (The Anchoveta Supply Chain project, ANCHOVETA-SC, http://anchoveta-sc.wikispaces.com).

**Table 2:** Fish to fishmeal and fish oil conversion ratios: national averages from 2001–2006.

Countries	Landings(1 000 t)	Fishmeal(1 000 t)	Fish oil(1 000 t)	FM ratios A	FO ratios	FM ratios B	Species used for reduction (common names)
Chile	3 161	773	157	4.09	20.13	3.93	Jack mackerel, *anchoveta*, sardine
China	2 041	769	-	2.65	-	3.80	Various
Denmark	881.5	327	106	2.70	8.32	3.01	Sandeel, sprat, blue whiting, herring
Iceland	1 262	221	74	5.71	17.05	4.44	Blue whiting, herring, trimmings
Japan	1 141	226	66	5.05	17.29	4.45	Sardine, pilchard
Norway	1 061	203	47.5	5.23	22.34	5.11	Blue whiting, capelin, trimmings
Peru	7 561	1 700	270	4.45	28.00	4.54	*Anchoveta*
**Peru (this study)^b^**	**6 498.2**	**1 551.4**	**320.6**	**4.21**	**21.30**		***Anchoveta***
United States	909	258	88	3.52	10.33	3.61	Menhaden, Alaska pollock

Notes: Landings, fishmeal (FM) and fish oil (FO) production, FM ratios A and FO ratios were taken from [122]. FM ratios B were taken from [123], for the period 2000–2005. Data for this study were taken from PRODUCE reported landings and production values for the period 2001–2011 [117].

### The processing industry for food

Peru surpassed 30 million inhabitants in 2012 [53], more than 70% of whom live in urban areas. Annual per capita fish consumption was estimated at ∼19 kg in 2005 and 23 kg in 2009. Consumption is notably higher along the coast (seafood) and in Amazonian areas (river fish), while it is much lower in the highlands (industrialised fish products and Andean aquaculture) [54].

The amount of fresh *anchoveta* landed for DHC has increased in the last decade at a mean annual rate of 37%, according to PRODUCE statistics. Nonetheless, DHC of only 1–2% of landings is low in a country with a large percentage of its population suffering from malnutrition [36]. It has been suggested that increased DHC of *anchoveta* could help solve some of the nutritional problems in Peru and the larger region [55].

Peruvian consumption of *anchoveta*, despite its recent increase, is still relatively small (3.3 kg per capita in 2010), yet it represents, on average, >70% of *anchoveta* DHC products. The scarcity of *anchoveta* for DHC is due to a combination of factors, including regulatory limitations (industrial vessels cannot supply the DHC industry), consumer preferences and lack of a cold chain for fish in Peru. Some believe a key factor is the shelf price of *anchoveta* DHC products. Moreover, one of the factors that direct or divert (for SMS captures) most *anchoveta* landings to reduction is the small difference, if any, in prices paid to fishermen per t of fish landed [32]. Fishmeal plants paid more than DHC plants until recently. Additionally, to keep *anchoveta* acceptable for DHC, vessels must carry ice, which reduces their holding capacity by at least 30%. These topics are further analysed in [32]. More detailed discussion of Peruvian *anchoveta* processing for DHC is presented in [56].

### Key *anchoveta*-based aquaculture systems in Peru

In Peru, aquaculture has been and is still dominated by scallops (*Argopecten purpuratus*) and shrimp (mainly *Litopenaeus vannamei*) for marine species and by trout (mainly *Oncorhynchus mykiss*), tilapia (*Oreochromis* spp.) and black pacu (*Colossoma macropomum*) for freshwater species [57], [58]. Marine aquaculture contributes ∼81% of Peruvian cultured fishfood production, while freshwater production represents ∼19% [59].

Peruvian aquaculture, mostly represented by small-scale or artisanal practices (∼63% of total production in 2010 [59]) has featured continuous growth over the last 20 years. Most trout culturing operations are artisanal yet semi-intensive, especially those in the Puno Department (Lake Titicaca and nearby water bodies), where most national production takes place. Trout farming in Puno department water bodies consist of artisanal wood- or metal-nylon floating cages (800–2000 kg carrying capacity) and larger metal-nylon floating cages (up to 6 000 kg carrying capacity). Trout is destined mainly for export, despite increasing consumption in the producing areas and larger cities of Peru, particularly Lima. Black pacu are cultured mainly in large, semi-intensive artificial pond systems, while tilapia is produced using a variety of methods and operational scales, mostly intensive. Black pacu is almost exclusively cultured in the Amazonia (Loreto and San Martin Departments) and tilapia in the Piura region. Black pacu is mostly consumed locally, mainly because of the physical isolation of the Amazonian communities that produce it. Tilapia was historically destined for national markets, but over the last decade increasing proportions of production have been exported.

Among these types of culture, shrimp aquaculture is the main consumer of fishmeal, given high percentages of fishmeal (20–50%) in commercial feeds [43], [60]–[62] and production volumes. As in other fish farming systems, a key aspect of Peruvian aquaculture is feed supply. In Peru, both artisanal and commercial feeds are used, but the latter prevail, especially for trout. National production of aquaculture products in Peru was estimated at 89 000 t in 2010, whereas national consumption was estimated at 0.52 kg per capita (∼15 000 t for a population of 29 million), yet a growth pattern in consumption of 22% per year has been recorded [59]. A more detailed discussion of Peruvian (freshwater) aquaculture is presented in [63].

### Distribution channels

Distribution channels for fisheries for DHC consist of 1) landing in several fishing ports and piers, both private and public; 2) transportation of fish in isothermal trucks, often organised by wholesalers; 3) processing in DHC plants; and 4) distribution to retailers for national consumption and export to foreign markets [64]. Most landing facilities for DHC have never met the requirements set by the sanitary standard for fisheries and aquaculture resources, as established by Supreme Decree 040-2001-PRODUCE [64]. The lack of a cold chain for fish in Peru is a major factor limiting further development of domestic distribution channels.

Peruvian aquaculture products are distributed within Peru by retailers (e.g. distributors, markets) and exported by producers or specialised exporting firms. Wholesaler markets concentrate ∼29% of total landings destined for fresh fish, 3.2% of which are not captured by Peruvian vessels but imported from neighbouring countries (mainly jack mackerel, *Trachurus murphyi*). In coastal areas, wholesaler markets supply retailers, supermarkets, restaurants and final consumers, although this does not apply to the scarce supply of fresh *anchoveta*. Lima alone accounts for 32% of national fish consumption.

Regarding canned fish, both processing plants and importers supply wholesalers, who subsequently supply supermarkets and retailers. Five percent of canned fish consumed in Peru is either imported as final product or as frozen fish to be processed in Peru, mainly tuna from Ecuador. Frozen food products are both produced in Peru and imported. Imports, representing ∼60% of frozen fish consumed in Peru, largely consist of jack mackerel (when national production of this highly fluctuating resource is too low) from Chile and tuna from Ecuador. Producers and importers supply wholesalers, who subsequently supply restaurants and supermarkets across the country (transported mainly in refrigerated trucks). Cured and salted products are both produced in Peru and imported, notably anchovy from Argentina (18% of national consumption of cured products). Producers and importers directly supply markets across the country.

### Fisheries management and policy environment

IMARPE provides the scientific foundation for fisheries management in Peru, which is implemented by PRODUCE [65]. IMARPE struggles between scientific and political considerations for its recommendations due to its relationship with PRODUCE (e.g. IMARPE’s Chairman of the Board is a political, rather than technical, position) [66].

IMARPE estimates the *anchoveta* population off Peru and recommends an annual TAC to PRODUCE [55]. This estimate is based on 1) hydro-acoustic data collected since 1975 from 2–3 annual surveys of the entire Peruvian coastline and 2) modelling of *anchoveta* population dynamics as a function of environmental conditions and recruitment levels using Virtual Population Analysis based upon a bio-economic age-structured model [67]. The recommended TAC is related to the Maximum Sustainable Yield. Spawning biomass is calculated using the Egg-Production Method (a meta-review is available in [68]).

Since the north-central stock contains >90% of the *anchoveta* biomass, most regulation and legislation applies only to it, leaving the south stock to be exploited under an open-access regime (featuring closures related to the proportion of juveniles in the total population). Fisheries legislation has been introduced since the early 1990s, and currently fisheries are mostly managed in an adaptive-reactive manner, with mixed effects. For instance, the decrease in catches to 3.4 million t in 2010 was due mostly to management measures applied to protect a large juvenile ratio. Because of that management decision, 2011 catches exceeded those of 2009 [12].

Other effects of legislation are still unfolding in the Peruvian *anchoveta* fishery and reduction industries. For instance, before 2008 legislation introducing individual vessel quotas (IVQ), up to 1200 vessels competed for the TAC in a so-called “Olympic race”, reducing the annual fishing season to 50 days [41], [69]. A list of key historical legislation governing fisheries in Peru is available in Table B1 in File S1. Fishing companies have reacted to the IVQ regime in various ways. For instance, large vertically integrated companies encompassing fishing and reduction are using their more efficient vessels to harvest their company-wide quotas (since IVQ are transferable within the same company) [41], [69]. As intended, this will eventually reduce fleet overcapacity, but has generated several other negative consequences [41], [70].

Most legislation regulates the activities of industrial, large-scale vessels, while the SMS fleets are poorly regulated and practically operate in an open-access regime [71]. Regulations on SMS fisheries include the exclusive use of the sea within 5 nautical miles (9.3 km) off shore, holding capacity, length, manual labour, mesh size of nets, prohibition of beach seines, minimum catch sizes for some species, and protection for cetaceans, turtles and seabirds [71], [72].

Some researchers consider that legislation related to Peruvian *anchoveta* is either insufficient, ineffective or poorly enforced [35], [41], [66], a situation affecting all *anchoveta* fleets. Moreover, several issues permeate the enforcement of Peruvian fisheries legislation and management guidelines (based on publications, pers. comm. with various researchers and experts, as well as on journalistic pieces), including the following:

- few data exist for smaller scale operations (Juan Carlos Sueiro, pers. comm., 2013)
- illegal, under-reported and un-regulated (IUU) landings are common [70]
- illegal reduction plants operate profusely, partially supplied by IUU landings (Pablo Echevarría, pers. comm., 2013)
- illegally produced fishmeal is “washed” by brokers
- regulations that mandate proper solid and liquid waste management from fishing vessels and processing plants are generally ignored
- capital and bargaining power are concentrated in a handful of vertically integrated companies
- SMS fisheries pay no fishing rights and have no quota assigned, while the money that industrial operations pay for fishery rights is clearly insignificant compared to their profits and insufficient to finance fishery regulation, supervision and control [47], [70], [73]–[76]

Despite these problems, Peruvian fisheries are generally considered among the most sustainably managed in the world [67], [77], [78], mostly because of their adaptive and reactive management measures that compensate for deficiencies in the legislation and management system. This management relies mostly on acoustic surveying-based annual quotas and on-demand fishery closures.

### Socio-economic dynamics

Fisheries and seafood products, especially exports of fishmeal and fish oil, represent the third largest individual source of foreign income for the Peruvian economy (on average, 8% from 2000–2011) [79]. China and Germany are the largest importers of Peruvian fishmeal, while Denmark and Chile are the main importers of fish oil. Most Peruvian fishmeal, most of which is high-quality, is destined for aquafeeds. In terms of employment, industrial and SMS fisheries, as well as reduction and other fish-processing industries, provide a large number of jobs. It is difficult to isolate the jobs associated exclusively with the extraction and processing of *anchoveta*, other than those in the reduction industries. Nonetheless, [80] estimated the number of jobs directly associated with the *anchoveta* industrial and SMS fleets at 10 000 and 8 000, respectively. Recently, employment in the Peruvian fisheries and processing sector was estimated more comprehensively [31]. These and other socio-economic indicators of *anchoveta* supply chains (gross profit generation, added value) are presented and discussed in [81].

### Nutritional value of fishfood products of *anchoveta* supply chains

According to the FAO and the Global Hunger Index [82]–[84], Peru has advanced in hunger reduction, yet remains one of the few Latin-American countries with moderate hunger. The International Food Policy Research Institute (IFPRI) defines “moderate hunger” as a level of hunger associated with a Global Hunger Index value of 5–10 out of 40. This index is built by combining three equally weighted indicators (undernourishment, child underweight and child mortality; as defined by FAO) [82], [84]. According to the FAO, hunger is associated with poverty [85]. Especially in Andean communities, indicators such as chronic malnutrition of children under five, stunting and undernourishment are still elevated [82], [85], [86], and thus government policies should be (and to some extent are being) oriented to provide these communities with cheaper sources of animal protein and improve access to nutritious food.

Seafood, especially that derived from the thriving *anchoveta* supply chains, has often been suggested as a suitable means to improve nutritional intake of vulnerable communities and people at large. Fishfood products of the *anchoveta*-based supply chains include *anchoveta* products as well as marine and freshwater aquaculture products. *Anchoveta* products are extremely high in beneficial omega-3 fatty acids, mineral salts and essential amino acids [55]. Further discussion on nutritional values of *anchoveta* and other Peruvian fishfood products is presented in [81].

### Ecosystem and bio-economic modelling of the Peruvian *anchoveta* fishery

The NHCS ecosystem and its sensitivity to environmental conditions, often emphasising population dynamics/stock assessment of commercially important species (e.g. *anchoveta* [87], [88] and Pacific hake (*Merluccius gayi*) [89] an *anchoveta* predator.) or threatened species (e.g. fur seals [90]), has been modelled since the 1970s [91], [92]. A preliminary EwE [17], [18] trophic model of the NHCS was presented in [93], highlighting that natural predators contribute more to total *anchoveta* mortality than fisheries. On the other hand, hake mortality, for instance, is due mostly to fisheries. A more comprehensive EwE-based trophic model was later presented by [94], [95], which discusses trophic and ecosystem dynamics under El Niño and La Niña conditions. The model by [94] was used to apply the ecosystem approach to hake and *anchoveta* fisheries [96], [97] and is currently used in the project IndiSeas [98]. Currently, these trophic models are not used for management because they are considered to be under development and to lack comprehensive data. Several bio-economic models have been also developed for the Peruvian *anchoveta* fishery [99], some of which have been used to estimate stock biomass and calculate the TAC. In recent years, new age-structured and integrated assessment models have been used by IMARPE [88].

### The proposed framework

The proposed framework is based on a one-way coupled model of the ecosystem and the supply chains that exploit it. It aims to provide tools and rationale for assessing and comparing current and future exploitation strategies of *anchoveta* and *anchoveta* supply chains by means of trophic, biophysical and socio-economic modelling.

### A one-way coupled ecosystem/supply chain model

**Figure 1:**
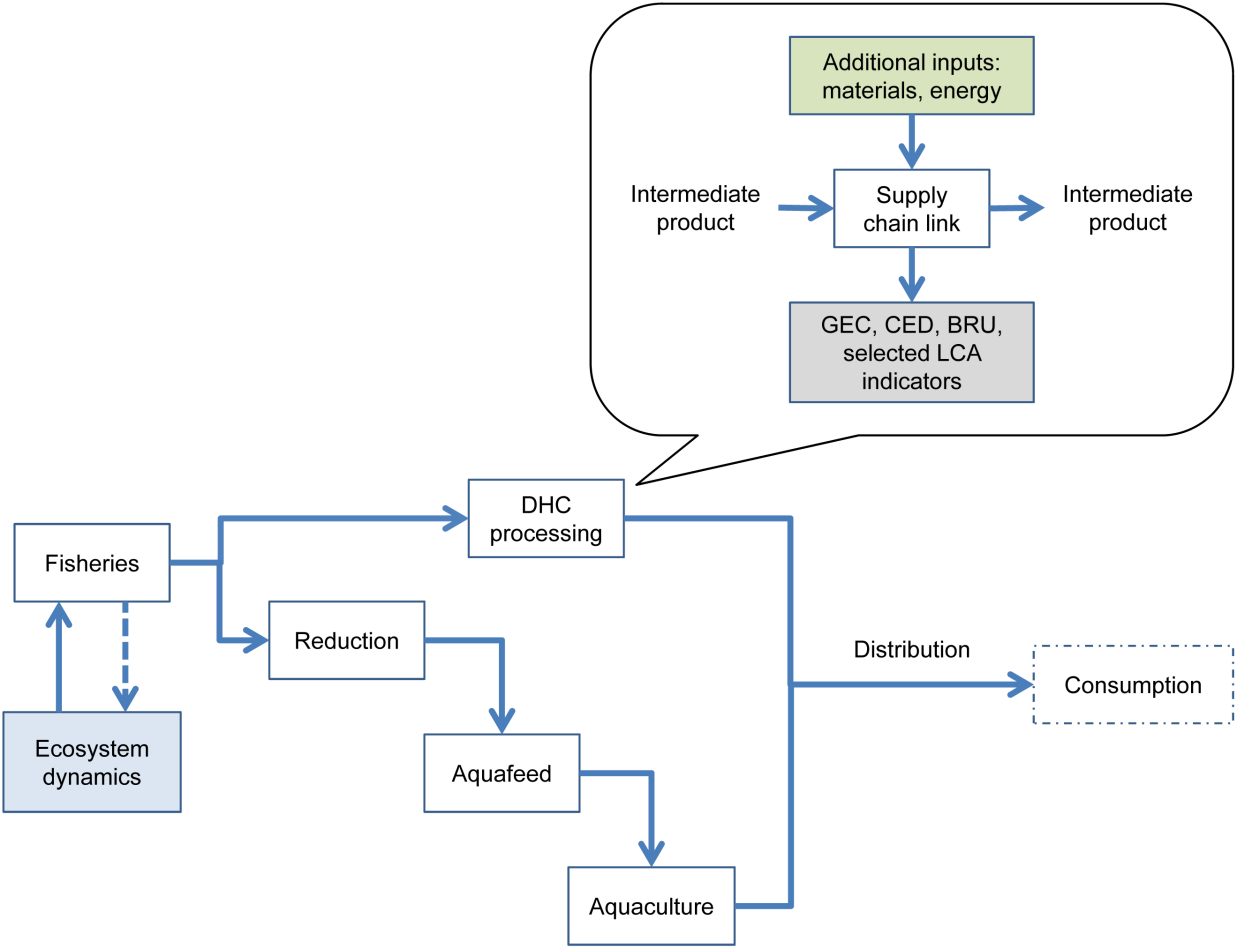
Simplified one-way coupled ecosystem/supply chain model. The zoom view illustrates how industrial processes and sub-processes are detailed within the supply chain. Environmental and socio-economic impacts of a given link of the supply chain are carried to the next link.

We propose an enlarged framework featuring an integrated ecosystem/supply chain model by combining existing models towards a holistic depiction of the ecosystem/seafood system interactions. This framework depicts flows and stocks of materials and energy occurring through the supply chain (from ecosystem to product retailing) and selected socio-economic elements (Figure 1). The proposed framework follows previous endeavours [29]–[31] in selecting EwE as a suitable ecosystem modelling platform to be coupled in a one-way or two-way manner with mass/socio-economic models. The frameworks differ in the approach for modelling supply chains. Our approach de-emphasises economic flows and highlights flows associated with the sustainability indicators selected to better describe sustainability performance of the system. The framework intends to assess overall sustainability, yet emphasises its environmental dimension, mainly due to data availability. We consider the proposed coupled model as an example of “ecosystem-based supply-chain modelling”. Moreover, the goals of both approaches differ as well: the value chain analysis in [30] accounts for socio-economic benefits of fisheries and subsequent links in the value chain, while our analysis compares the relative sustainability performance of competing fisheries-based supply chains.

In our framework, monetary flows are analysed at the industrial-segment level rather than at the value chain level; that is to say, no individual firms are modelled, but rather whole production sectors (e.g. fisheries, reduction industry, species-specific aquaculture sector) by aggregating and generalising individual firm results.

An EwE trophic model of the marine ecosystem exploited by the modelled supply chain can be used as the base ecosystem model. The outputs of the EwE model would feed a material and energy-flow model, which could be built, for example, with Umberto, a modelling tool specifically designed to study material flow networks [100]. Umberto represents material flow networks as Petri nets, that is to say, in terms of transitions (transformational processes), places (placeholders for materials and energy) and arrows (flows). These are the modelling tools/approaches that we selected, but almost any combination of a whole-ecosystem model and a MFM would be suitable, especially if the coupling could be established in a dynamic fashion (i.e. models interacting in real time during simulations).

**Figure 2:**
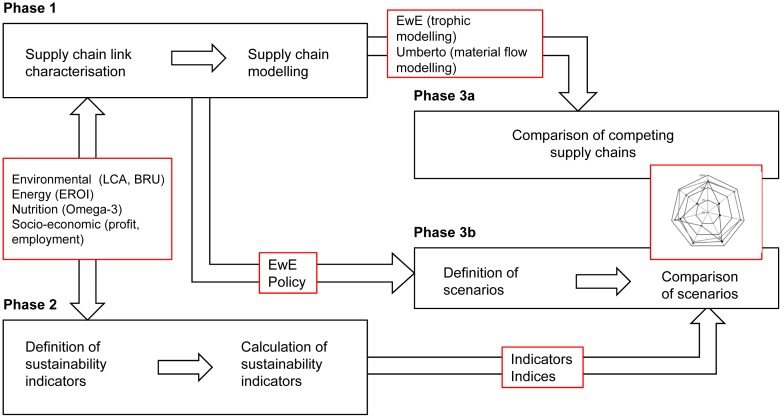
Proposed sustainability assessment framework for seafood supply chains.

The framework has three main phases (Figure 2): 1) characterisation and modelling of the fishfood system under study, 2) definition and calculation of sustainability indicators, 3a) comparison of competing supply chains, and 3b) definition and comparison of alternative policy-scenarios for the set of all supply chains. Phases 1 and 2 are to a certain extent concurrent, since the selection of sustainability indicators largely determines the direction and complexity of system characterisation (data collection and processing).

In Phase 1, material, energy, nutritional and monetary flows of target supply chains, both short (DHC products) and long (aquaculture), are modelled. In Phase 2, a set of suitable sustainability indicators is compiled to compare the performance of supply chains modelled in Phase 1, as detailed and illustrated for a subset of *anchoveta* supply chain-derived products in [81]. In Phase 3, supply chains are compared and policy-based scenarios for future exploitation and production are defined and contrasted.

Since the main goal of the characterisation stage is to inform sustainability assessment of complex anthropogenic systems directly interacting with ecosystems, the characterisation must include both biophysical and socio-economic flows. The study of biophysical flows illustrates ecosystem/industry interactions and provides data about flows and stocks of materials and energy occurring along the supply chain, including their effects on the environment. In contrast, analysis of socio-economic flows offers insights about social and economic dynamics occurring parallel to the material ones. By understanding the system from at least these three perspectives, sustainability can be evaluated.

### Supply chain characterisation and modelling

The biophysical accounting framework used to model supply chains was Life Cycle Assessment (LCA). LCA is a mature approach, and current Life Cycle Impact Assessment (LCIA) methods encompass a great diversity of environmental impact categories. Socio-economic aspects would ideally be assessed by combining life cycle methods and economic analysis frameworks, such as Life Cycle Costing (LCC), Social LCA and cost-benefit analysis. Nonetheless Social LCA is not yet mature, and it is usually difficult to obtain all the data required from fishery and fishfood industries to apply it [101], [102]. Not enough data were available for LCC or cost-benefit analyses.

Several LCA studies were required to characterise environmental impacts and resource consumption (including energy use) of components of fish supply chains: fisheries, processing for DHC, reduction into fishmeal and fish oil, aquaculture and distribution. LCAs were performed using the software SimaPro [103], which features integration with the widely used database ecoinvent [104] and various LCIA methods, including CML baseline 2000 [105], ReCiPe [106], Cumulative Energy Demand [107] and USEtox [108]. LCA methodology and results associated with *anchoveta* supply chains are presented in [39], [40], [42], [56], [63].

LCA results (including additional and fishfood-specific impact categories and other Life Cycle Inventory-based indicators), EwE outputs and socio-economic performance indicators become inputs for the Umberto modelling environment. Umberto outputs include mass and energy balances and flow diagrams (e.g. Sankey diagrams).

### Definition and calculation of the indicator set

Once the target supply chains are modelled based upon detailed operational and socio-economic data, a set of sustainability indicators is calculated to assess sustainability and compare alternative supply chains (e.g. DHC vs. IHC chains based on the same fishery).

Several sustainability indicators were selected from the large indicator pool available in the literature so that all aspects of sustainability –especially the environmental dimension, but also energy efficiency, human nutrition and socio-economic factors– were addressed. Main criteria for this selection were historical use in the fishfood research field; purpose (mainly environmental plus key socio-economic aspects); practicability, given data availability; and comparability with other food systems. Table 3 lists the indicator set, introduced and detailed in [81], and expanded in this study with a few IndiSeas ecological indicators [109], [110] to compare alternative states of the exploited ecosystem. The indicators “Trophic level of landings”, “Proportion of predatory fish” and “Inverse fishing pressure” can be used to measure maintenance of ecosystem structure and functioning, conservation of biodiversity and maintenance of resource potential, respectively (Eq. 1, 2 and 3 [110]):

**Table 3:** The sustainability indicators proposed, per dimension of sustainability addressed.

Sustainability dimension	Indicator (unit)	Reference publications	Calculation
Ecological	*I_BNR,sp_* (years)	[124]	Manual
	*I_BNR,eco_* (years)		
	TL_land_	[110]	
	Proportion of predatory fish		
	Inverse fishing pressure		
Ecological/environmental	BRU (g C kg^−1^)	[125]	Manual
	BRU-based discard assessment	[126], [127]	
Environmental	LCA/ReCiPe (Pt)	[128]	LCIA methods
	LCA/CED (MJ)	[107]	
	LCA/CML [USES-LCA] (kg 1,4-DB eq)	[105], [129]	
	LCA/USEtox (CTU)	[108]	
Nutritional	GEC (MJ kg^−1^)	[130]	Manual
	Nutritional profile	[131]	
Energy efficiency	Gross edible EROI (%)	[130], [132], [133]	Manual
	Edible protein EROI (%)		
Socio-economic	Production costs (USD)	[134]	Manual
	Employment (USD)		
	Value added (USD)		
	Gross profit generation (USD)	Accounting concept	

Abbreviations: BRU: Biotic Resource Use, CED: Cumulative Energy Demand, CTU: comparative toxic units, EROI: Energy Return On Investment, GEC: Gross Energy Content, *I_BNR,sp_*: impacts on Biotic Natural Resources at the species level, *I_BNR,eco_*: impacts on Biotic Natural Resources at the ecosystem level, LCA: Life Cycle Assessment, LCIA: Life Cycle Impact Assessment, TL_land_: Trophic level of landings. Modified from [81].



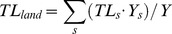
(1)where *TL* is the trophic level, 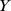 is catch and *s* is species.



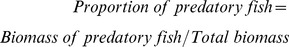
(2)where *Total biomass* includes the biomass of demersal, pelagic and commercially relevant invertebrates.



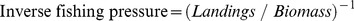
(3)where *Landings* and *Biomass* refer to those of the species selected. For these three indicators, a larger value represents in principle a healthier ecosystem (but see discussion).

### Definition and comparison of policy-based scenarios

**Figure 3:**
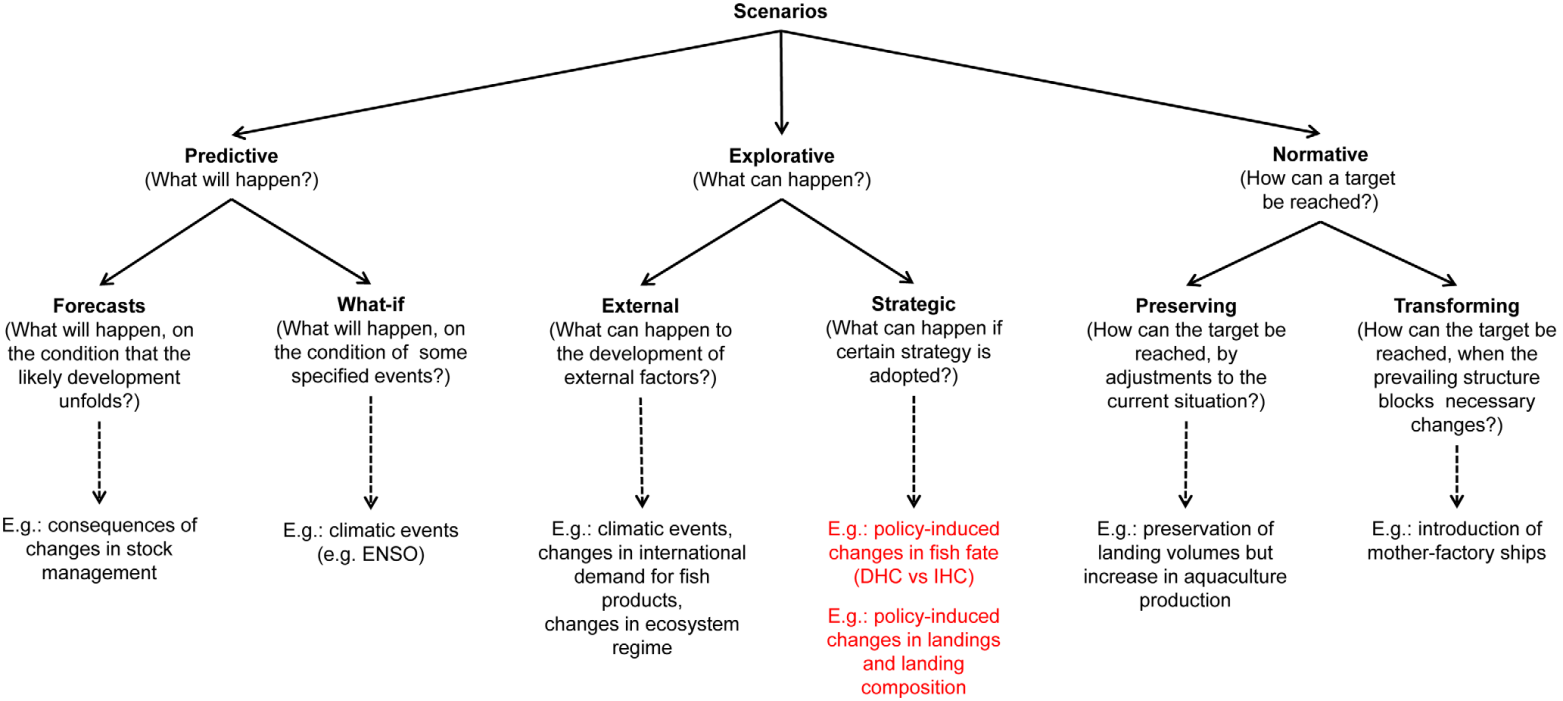
Types of scenarios suitable for seafood sustainability research. Based on [111]. Examples in red represent the preferences of this research. DHC: direct human consumption; IHC: indirect human consumption (i.e. reduction).

In the context of fishfood research, comparing the sustainability of competing or alternative exploitation scenarios could inform decision making. Figure 3 illustrates proposed scenarios for comparing sustainability of fishfood supply chains, using the typology discussed in [111] (see also section A in File S1).

By integrating the ecosystem compartment in the supply chain model, it is possible to predict, for instance, changes in stock related to changes in exploitation regimes. Changes in stock (e.g. stock recovery) are not only linked to fishing pressure, but also to ecological processes [16]. The EwE model features biological processes such as respiration and predation. It can represent environmental regime shifts and ENSO events as different scenarios (e.g. states of the NHCS in an El Niño and non-El Niño year [94]), although this was not explored here. The integration can also help estimating overall environmental impacts associated with alternative fates of landed fish.

Supply chains and policy-based scenarios are compared based on functional units, typically one t of fish (live weight) produced or processed. Supply-chain-wide flow analyses and product comparisons by means of the sustainability indicator set are the comparison tools. Visualisation devices include mass and energy balances, tables, Sankey diagrams [112], [113] and graphs.

### Data sources and methods

Establishing inventory data for LCAs was the most data-intensive endeavour in this study. Most background processes had been previously modelled in ecoinvent and reference publications. Data were collected in Peru from 2008–2013 in the ANCHOVETA-SC project, in cooperation with PRODUCE, IMARPE, the Research Institute of the Peruvian Amazonia [114], a trout development project from the Puno regional government [115], Peruvian universities, various large fishing and reduction enterprises –organised into the National Fisheries Society [51]–, as well as many confidential and anonymous sources. Detailed statistics and operational data about all key links in the complex *anchoveta*-based supply chains were gathered. Moreover, experts and analysts of the *anchoveta* industries were also approached, and historical datasets obtained from them, some including data from a large enterprise no longer in operation but whose vessels were operated by other companies. Surveys were extensively used to obtain data, particularly from industrial and SMS fisheries. Field visits included fishing ports, fishmeal plants, fish processing plants, aquaculture farms and shipyards. Details about all data sources used are presented in [39], [40], [42], [56], [63], [81].

A screening-level LCA (Life Cycle Screening, LCS) of the industrial hake fleet was performed using literature data and landings statistics from PRODUCE and IMARPE (R. Castillo, pers. comm., 2013; R. Adrien, pers. comm., 2013). This screening relied heavily on assumptions, since detailed data on Peruvian hake fisheries was not available. Based on these uncertain data, sustainability indicators were calculated so as to compare the fishery of this carnivorous fish with those of *anchoveta* and another carnivorous fish (farmed trout), as well as their respective products.

The ecosystem model used is based on the above-mentioned EwE trophic models of the NHCS by [94], [95]. The model domain extends from 4°–16° S and 60 nautical miles (111 km) offshore, covering an area of ∼165 000 km^2^ and including 32 living functional groups. The model was fitted to historical time-series data of biomass and catch of main fishery resources from 1995–2003. After the historical period, scenario simulations were run for the period 2004–2033. A key feature of the EwE scenarios was the behaviour of *anchoveta* and hake biomasses. Observed and fitted *anchoveta* biomasses decreased during El Niño in 1997–1998, recovered in 2000, and fluctuated until stabilising around 70 t km^−2^. On the other hand, hake biomasses also decreased during El Niño, but recovered more slowly in 2006 and stabilised around 1 t km^−2^.

**Figure 4:**
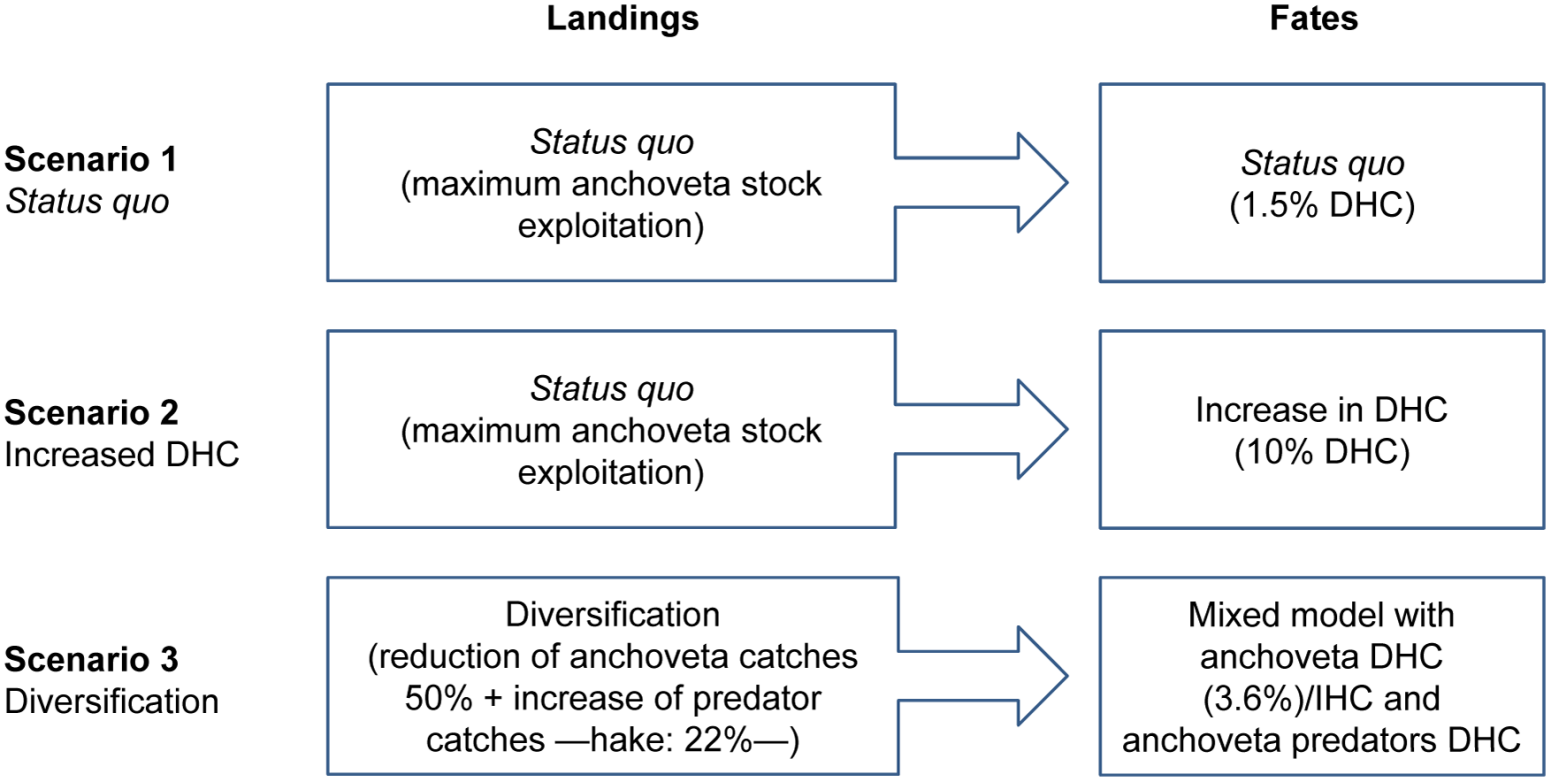
Alternative exploitation scenarios.

Figure 4 lists the alternative exploitation scenarios derived from the EwE simulation. These scenarios were recommended in [32]. Two types of scenarios seemed suitable, both policy-induced: 1) changes in fish fates (DHC vs. IHC) and 2) changes in landings and landing composition. Therefore, three alternative exploitation scenarios were derived from the EwE model, projecting the reference year (2011) into the future:

- Scenario 1 (S1) - *Status quo*. This is an extrapolation of the current situation (2011) in which the *anchoveta* fishery is fully developed and landings oriented to DHC remain low, varying from 1.5% in the reference year 2011 to 3.6% in 2021. The increase in the percentage of DHC represents an extrapolation of the current slightly increasing trend. After the historical period, *anchoveta* and hake fishing mortality are set constant and equal to the last historical value.
- Scenario 2 (S2) - Increased DHC. The same fully developed *anchoveta* fishery as in Scenario 1, but 10% of the landings are oriented to DHC. *Anchoveta* and hake fishing mortalities are the same as in Scenario 1.
- Scenario 3 (S3) - Diversification. In this scenario, *anchoveta* exploitation decreases and exploitation of hake, increases. *Anchoveta* landings are oriented both to DHC and IHC, and hake landings are oriented to DHC. From the end of the historical period onwards, *anchoveta* fishing mortality was linearly decreased to 50% over the next ten years (2013), then hake fishing mortality was linearly increased to 22% over the next ten years (2023); afterwards, fishing mortalities were kept constant for 10 more years (2033) to stabilise EwE outputs.

EwE modelling provides the ecosystem perspective of these scenarios, while LCA-derived and other indicators based on a functional unit can easily be scaled up or down to varying production volumes. The one-way coupling between the EwE model and the MFM, built with Umberto [100], is mono-directional, since dynamic linking was not feasible. EwE outputs are inputs to the MFM model, but changes in the MFM model cannot influence the EwE model directly. Therefore, the one-way coupled model was used to model the current situation and alternative fish-exploitation scenarios. Nonetheless, the MFM model can be used alone, as a supply chain modelling tool to explore variations within a defined scenario (e.g. changes in relative production volumes of aquaculture products or *anchoveta* DHC products). The Umberto project file containing the MFM model is available upon request to the corresponding author.

For the alternative exploitation scenarios, changes in the percentage of *anchoveta* landings destined to DHC and in aquaculture production were modelled for future years by extrapolating historical landing and production data [116], [117] using statistically representative trend lines. Operational costs and prices were not extrapolated due to a lack of detailed annual data. Eventual changes in captures per unit effort (CPUE), which is accepted to be proportional to changes in biomass and fish catchability (affecting fuel-use intensity), were considered, in such a way that all environmental modelling in this study is based on CPUE-adjusted fuel use intensities.

The coupled trophic/supply chain model is fed from several models: the EwE trophic model of the NHCS, LCAs of each link in the *anchoveta* supply chain, and additional sustainability and nutrition indicators (Table 4).

**Table 4:** Modelled sub-systems of the Peruvian anchoveta supply chain.

		Biophysical indicators				
Sub-models →	EwE outputs	LCA	LCS	Other environ-mental indicators	Nutrition/energy indicators	Socio-economic indicators
Supply chain links ↓						
**Fisheries**						
Industrial *anchoveta* fleet	X	X		X	X	X
Vikinga (*anchoveta*) fleet	X	X		X	X	X
Small- and medium-scale (SMS) *anchoveta* fleet/average landed *anchoveta* for IHC	X	X		X	X	X
Average landed *anchoveta* for reduction (weighted mean of industrial and Vikinga fleets)	X	X		X	X	X
Ice plants supplying SMS fisheries			X			
Industrial hake fishery	X		X	X	X	
**Direct Human Consumption**						
Canned *anchoveta*		X		X	X	X
Frozen *anchoveta*		X		X	X	X
Salted/cured *anchoveta*		X		X	X	X
**Indirect Human Consumption (reduction)**						
Prime fishmeal		X		X	X	X
Fair Average Quality fishmeal		X		X	X	X
Residual fishmeal			X	X	X	X
**Aquafeeds**						
Artisanal feeds, Peru		X		X	X	X
Commercial feeds, Peru			X	X	X	X
Commercial feeds international (ingredients and energy use)			X	X	X	X
**Aquaculture**						
Tilapia: artisanal/commercial feeds, Peru			X	X	X	X
Black pacu: artisanal/commercial feeds, Peru		X		X	X	X
Trout: artisanal/commercial feeds, Peru		X		X	X	X

Abbreviations. LCA: Life Cycle Assessment, LCS: Life Cycle Screening, IHC: Indirect Human Consumption.

## Results

### Comparison of current supply chains

**Figure 5:**
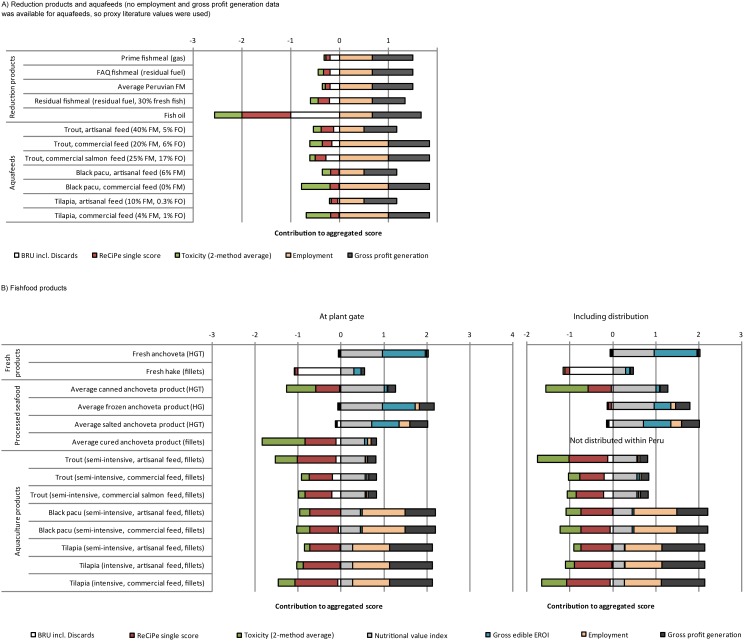
Ranking of DHC products studied from *anchoveta* supply chains according to the proposed indicator set. Per t of fish in product. Shorter negative bars and longer positive bars represent better performance: the range of values on the x-axis represents the maximum positive and negative scores possible for each product. In bottom graphs, all units have the same length, even though the x-axis of the right-side graph was shortened for convenience. Only five indicators are shown in order to limit redundancy between indicators, simplify the diagram, and increase balance among indicators from the three pillars of sustainability (impacts are cumulative and no weighting factor was used). Species names: anchoveta (*Engraulis ringens*), black pacu (*Colossoma macropomum*), hake (*Merluccius gayi*), trout (*Oncorhynchus mykiss*), tilapia (*Oreochromis* spp.).

The proposed ecosystem/supply chain model produced an overview of the sustainability of the entire *anchoveta* supply chain. The MFM is presented in Figure B2 in File S1. All studied products were ranked (Figure 5), including distribution at the national level of fisheries-DHC and aquaculture products. Fresh *anchoveta* and low energy-intensive *anchoveta* products perform better from a sustainability perspective than other products. [81] presents a more detailed comparison of *anchoveta* DHC and aquaculture products, representing the current status of these supply chains.

When including national distribution of DHC products (using refrigerated chains when necessary), overall environmental performance (represented by the ReCiPe single score and toxicity indicators) increase, with a wide range of values (from 3% for canned products to 250% for frozen products). Nonetheless, the relative environmental ranking of studied products does not change significantly, because distribution contributes relatively little to total impacts (Table 5).

**Table 5:** Comparison of environmental performance of fisheries and aquaculture direct-human-consumption products, at plant gate and after distribution, per t of fish in product.

		At plant gate			Including distribution			Percentage increase	
Product group	Products	ReCiPe singlescore (Pt)	Toxicity (CML,kg 1,4-DB eq)	Ranking (1 = best)	ReCiPe singlescore (Pt)	Toxicity (CML,kg 1,4-DB eq)	Ranking(1 = best)	ReCiPesingle score	Toxicity (CML)
Fresh products	Fresh *anchoveta* (HGT)	31	51 918	1	51	75 829	1	68%	46%
	Fresh hake (fillets)	111	129 869	4	205	241 039	4	85%	86%
Processed seafood	Average canned *anchoveta*product (HGT)	866	3 229 195	6	893	3 260 146	5	3%	1%
	Average frozen *anchoveta*product (HG)	38	60 272	2	132	171 443	3	250%	184%
	Average salted *anchoveta*product (HGT)	46	103 633	3	62	122 566	2	36%	18%
Aquaculture products	Trout (semi-intensive,artisanal feed, fillets)	1 045	1 783 975	8	1 140	1 895 146	8	9%	6%
	Trout (semi-intensive,commercial feed, fillets)	849	1 151 958	5	943	1 263 129	6	11%	10%
	Trout (semi-intensive,commercial salmon feed, fillets)	980	1 170 740	7	1 074	1 281 910	7	10%	9%
	Black pacu (semi-intensive,artisanal feed, fillets)	1 052	1 158 268	10	1 146	1 269 438	10	9%	10%
	Black pacu (semi-intensive,commercial feed, fillets)	1 045	1 121 131	9	1 140	1 232 301	9	9%	10%
	Tilapia (semi-intensive,artisanal feed, fillets)	1 105	1 017 474	11	1 200	1 128 644	11	9%	11%
	Tilapia (intensive,artisanal feed, fillets)	1 355	1 178 435	12	1 450	1 289 605	12	7%	9%
	Tilapia (intensive,commercial feed, fillets)	1 573	1 653 337	13	1 667	1 764 507	13	6%	7%

Abbreviations. HGT: headed, gutted, tailed.

**Figure 6:**
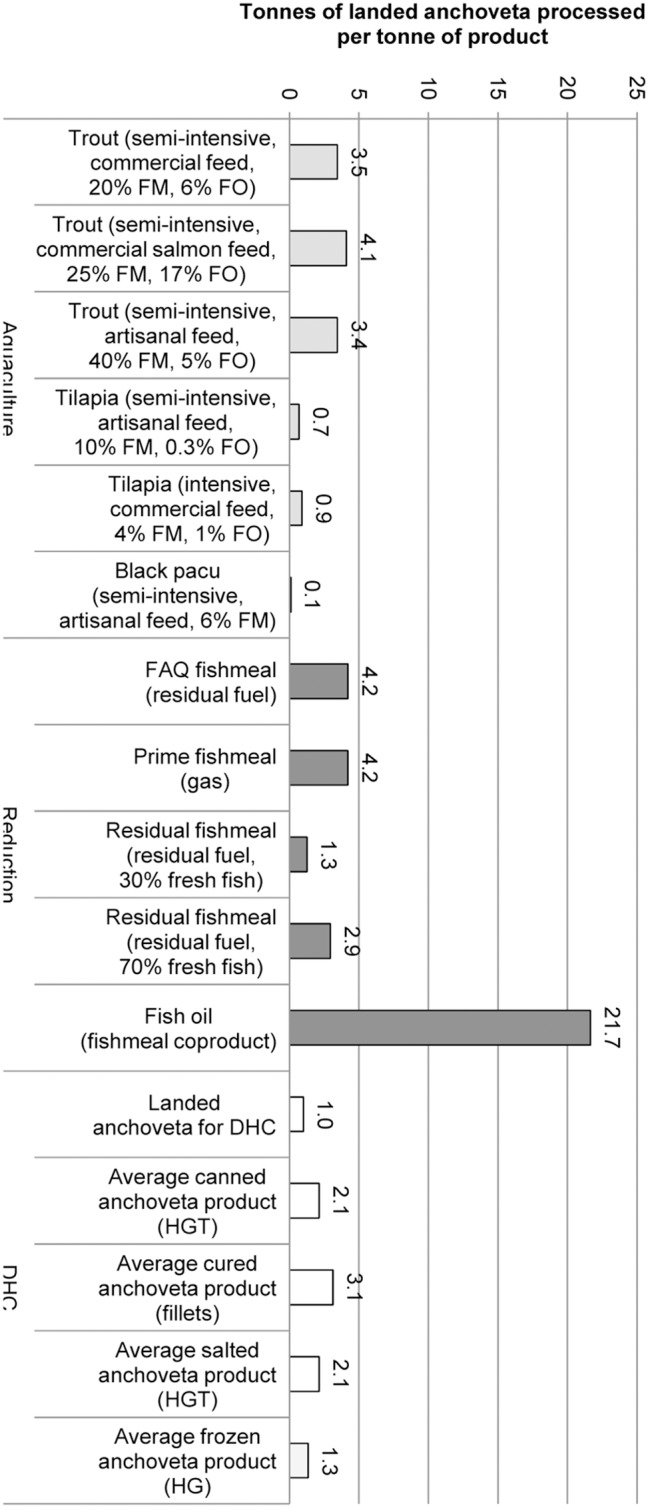
Alternative fates of 1 t of landed *anchoveta*. Excluding other agricultural inputs to aquafeeds and DHC products, expressed as tonnes of landed anchoveta processed into 1 t of final product; HGT: headed, gutted, tailed; FM: fish oil. Species names: anchoveta (*Engraulis ringens*), black pacu (*Colossoma macropomum*), trout (*Oncorhynchus mykiss*), tilapia (*Oreochromis* spp.).

The fate of one t of Peruvian *anchoveta*, from sea to plant or farm gate (and to port gate for fresh *anchoveta* for DHC) was calculated (Figure 6). DHC products have markedly higher yields of products (and are directly edible by humans) than reduction products. Aquaculture products are not directly comparable because they also require agricultural inputs.

### Alternative exploitation scenarios

In S1 and S2, *anchoveta* biomass (Figure C3 in File S1) and hake biomass (Figure C4 in File S1) remained stable in the simulation based on historical values because no further changes were introduced. However, in the diversification scenario (S3), due to the decrease in *anchoveta* landings, *anchoveta* biomass increased by 21% and stabilised around 85 t•km^−2^ (Figure C3 in File S1). Consequently, hake biomass increased by 18% and stabilised around 1.2 t•km^−2^ (Figure C4 in File S1). It is noteworthy that biomasses of other predators also increased in this scenario (e.g. other piscivorous fish such as Eastern Pacific bonito (*Sarda chiliensis chiliensis*), seabirds and pinnipeds), yet hake is the most commercially interesting species among them. EwE outputs for 2011 and simulation scenarios, including fish biomasses, are presented in section C in File S1. A key input datum for the hake fisheries LCS is mean fuel-use intensity, estimated at 84 kg fuel per landed tonne, mass-allocated between hake and by-catch (93% of landings were hake, according to detailed landing records for the hake fleet in 2010; IMARPE, unpublished data).

**Figure 7:**
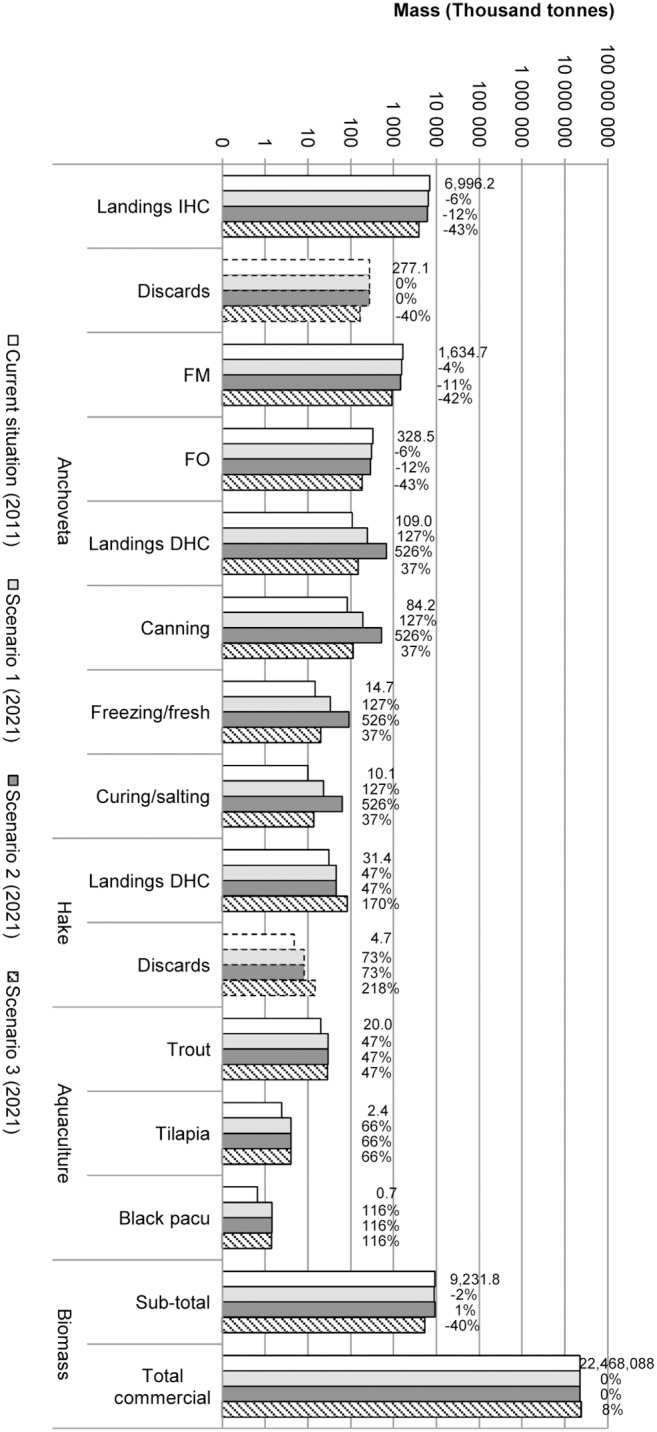
Mass outputs associated with alternative exploitation scenarios. Per key product on a log_10_ scale. Percentages represent variation from the current situation. Species names: anchoveta (*Engraulis ringens*), black pacu (*Colossoma macropomum*), hake (*Merluccius gayi*), trout (*Oncorhynchus mykiss*), tilapia (*Oreochromis* spp.).

From the main masses of products in the three scenarios in the reference future year 2021 (Figure 7), conclusions about masses of target seafood products and the total biomass of all commercial species in the marine ecosystem can be drawn: the former increases by 1% in S2 and decreases by 40% in S3, while the latter does not change in S2 and increases by 8% in S3. Sankey diagrams [112], [113] of the main masses (biomass and other materials) and energy flows were produced for the supply chains in the reference year 2011 and for the three scenarios in 2021 (Figures B3 to B5 in File S1).

**Figure 8:**
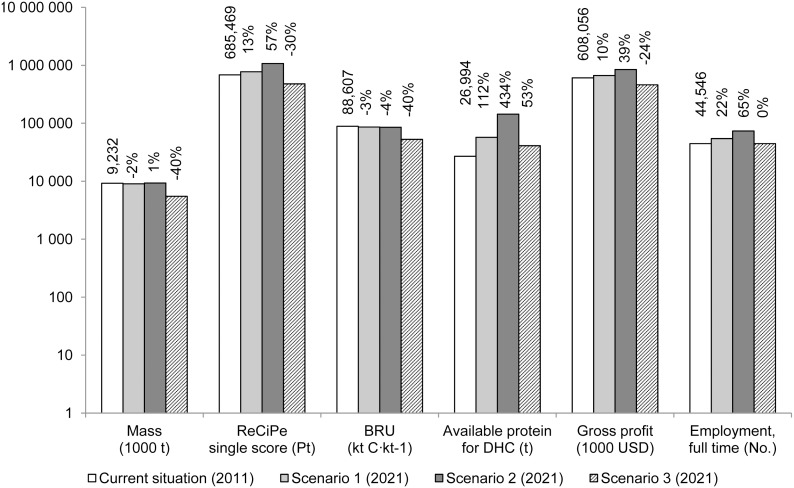
Comparison of alternative exploitation scenarios. In terms of product masses, environmental score, biotic resource use, human nutritional protein availability, gross profit and employment per key product on a log_10_ scale. Percentages represent variation from the current situation.

Graphical comparison of the scenarios according to other dimensions of analysis (e.g. ecological and socio-economic) is presented in Figure 8 (and detailed per product in Figures B6 to B10 in File S1). The results depicted in these figures refer only to the fishfood products studied.

Comparative gross economic benefits are expressed as gross profit (revenues – production costs). Gross profit of the fishfood-product supply chains studied increases by 12% in S2 but decreases by 36% in S3. Detailed mass and economic balances, as well as detailed data for other dimensions of analysis (environmental impacts, biotic resource use, nutritional value) are shown in Tables B2 and B3 in File S1. Employment related to the fishfood-product supply chains studied increases by 18% in S1, which was expected due to the increase in job-intensive production of DHC products. In S2 the increase in employment reaches 53%, while in S3 employment decreases by 6%.

Environmental impacts, as expressed by the ReCiPe single score, increase by 10% in S1 and by 54% in S2, associated with increased production of energy-intensive processed seafood products. In S3, environmental impacts decrease by 32% due to the large decrease in *anchoveta* landings. The biotic resource use subtotal decreases by only 3% in S1 and 4% in S2, but decreases by 40% in S3, also due to the large decrease in *anchoveta* landings.

The sum of available protein (a proxy for the nutritional value of each scenario) of target products increases in all scenarios from 2011 to 2021. In S1, the 112% increase in the available protein of target products is associated with increasing landings for DHC, while in S2 the increase is 434%. In S3, the increase, by comparison, appears moderate (53%) but substantial due to the increase in hake landings for DHC. It is worth noting that the sum of available protein of other commercial species such as Peruvian sea catfish (*Galeichthys peruvianus*), fine flounder (*Paralichthys adspersus*) and Eastern Pacific bonito, display a different pattern from that for target products, with small decreases of 4% from 2011 in S1 and S2 but a 47% increase in S3. When this increase is expressed as an absolute value (2 039 Mt) it overcompensates the lower available protein subtotal in S3 compared to those of S1 and S2 (−2.2 Mt).

**Figure 9:**
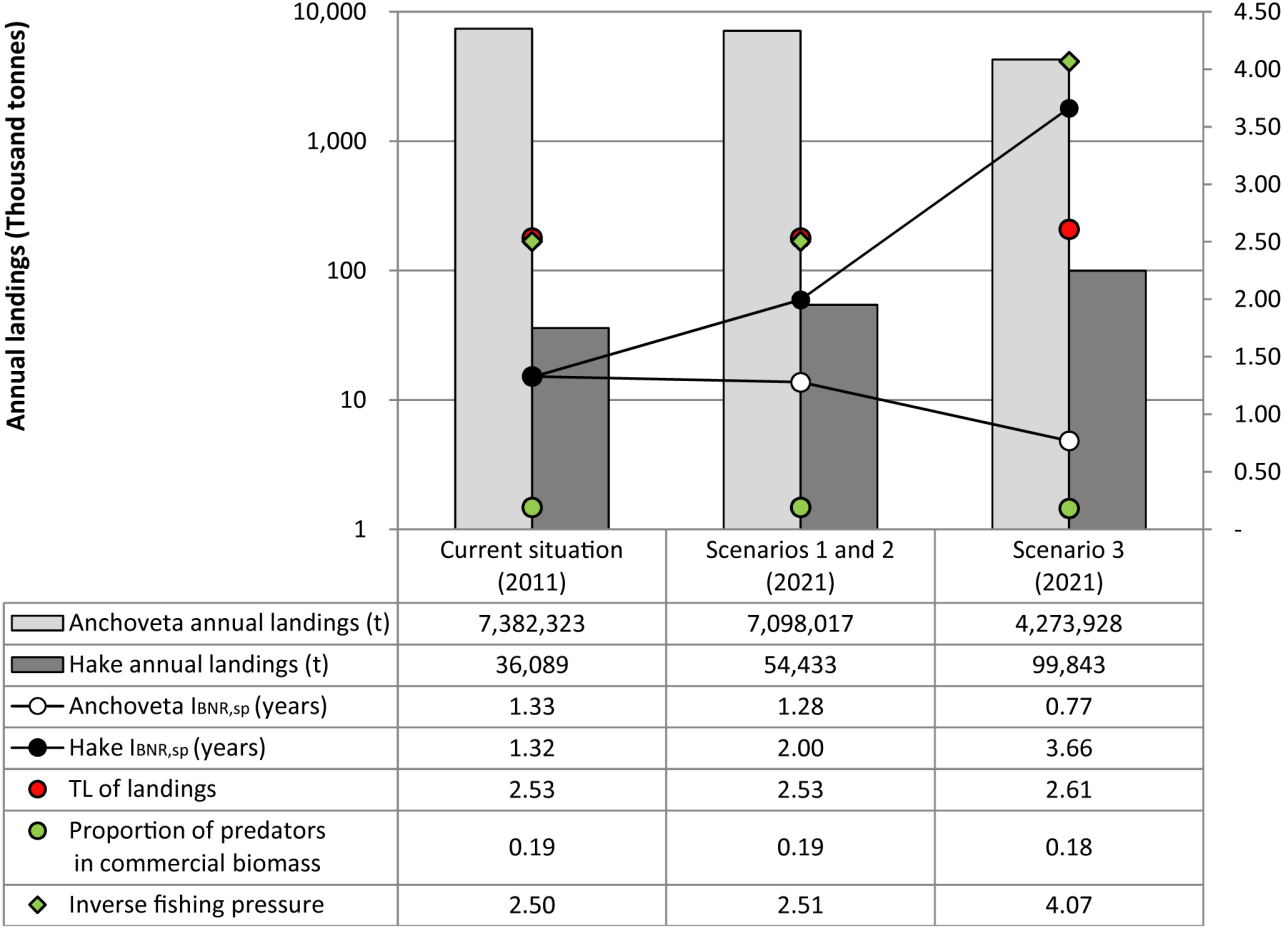
Indicators of ecosystem impacts. Impacts on Biotic Natural Resources (I_BNR_) at the species level, mean trophic level (TL) of landings, proportion of predatory fish in commercial biomass, and inverse fishing pressure under the alternative exploitation scenarios. The Maximum Sustainable Yield (MSY) of *anchoveta* was estimated at over 5 million t [135]; thus, a 5-year mean of total landings (5.5 million t) was used as a proxy. The MSY of hake was estimated at ∼27,000 t until its stock fully recovers [89]. Species names: anchoveta (*Engraulis ringens*), hake (*Merluccius gayi*).

Among the ecosystem level indicators chosen (Figure 9), a higher value for I_BNR,sp_ represents lower ecosystem health, while the higher values for all IndiSeas indicators represent a healthier ecosystem. Results of I_BNR,sp_ among scenarios (the same amount of biomass is extracted in S1 and S2) show progressive improvement for *anchoveta* and worsening for hake. Applying IndiSeas indicators to EwE outputs of all commercial species results in an increase in the trophic level of landings, from 2.53 in S1 and S2 to 2.61 in S3. They also show an increase in inverse fishing pressure from 2.51 to 4.07, while the proportion of predatory fish decreases slightly in S3, from 0.19 to 0.18.

## Discussion

### Methodological choices

The use of trophic level (TL)-based ecological indicators is suitable for Peru because its fisheries are fully- or over-exploited. TL-based indicators, under the fishing-down-the-food-web concept [118], represent a measure of ecosystem structure and functioning and thus can be used to measure state and trends. These indicators would be less useful in situations in which exploitation is still developing.

Both 1) the proportion of *anchoveta* landings destined for DHC and 2) aquaculture production are expected to grow. Complete historical annual data was available until 2011, and both DHC and aquaculture production datasets indicated a growing trend until 2010. Nonetheless, in 2011 total *anchoveta* landings for DHC were lower than those in 2010, but it was not possible to predict a decreasing trend based on a single “low-catch” year. Aquaculture output, on the other hand, shows continuous growth since 2001.

If future scenarios had been built assuming no growth, relative results would not differ significantly. Particularly in the case of S3, in which total *anchoveta* landings dramatically decrease, we simulated the fate of *anchoveta* landings maintaining the trend of DHC of the reference situation (2011). That is to say, the landing ratios of S1 (∼3.6% to DHC) were kept. Since reduction and canning industries are highly vertically integrated and have overcapacity, it is likely that a shortage of *anchoveta* would severely constrain fish reduction, leading firms to prioritise their most recent investment: processing of *anchoveta* for DHC, especially canning. The fact that operational costs and prices were not extrapolated is not a major issue, since our approach is comparative.

Another fundamental decision for scenario modelling was that the reference situation (2011) was modelled (in the MFM) using biomasses from PRODUCE statistics rather than from EwE predictions. Differences are minor, but we preferred the more realistic depiction of the reference situation. For future scenarios, total catches (resulting from the fishing mortality rate) for *anchoveta* and hake were taken from EwE predictions, as previously described, but the uncertainty in predictions of this kind of model [21], [119] was not considered. As a result, predictions for future scenarios must be used with caution and regarded as tentative indications of trends. Reduction efficiencies were not altered (we assumed that the technical optimum has been reached), nor were aquaculture data (e.g. inter-species production ratios, general trends in feed compositions).

### The current situation: could it be better?

In the current situation, a variety of *anchoveta*-based products are produced. The fishmeal industry has improved its technical performance over the years, and the current state-of-the-art mainly involves use of natural gas and an indirect drying process. Prime-quality fishmeal produced at gas-based indirect drying plants has the best sustainability performance according to the set of sustainability indicators applied. Nonetheless, the legal production of residual fishmeal remains necessary, not only from a socio-economic standpoint, but also from the environmental standpoint, to make the best use of fish resources.

As for DHC products, optimum sustainability would come from landing, processing and distributing fresh/chilled/frozen *anchoveta* products; however, salted and canned products currently provide certain vulnerable communities with fish products. Freshwater aquaculture products could play a larger socio-economic role in Peru if an adequate distribution chain is established and current landing infrastructure for the SMS fleet is improved and enlarged. Among cultured species, black pacu has higher sustainability performance. Moreover, production of black pacu (and by extension other Amazonian species) seems promising for Peru, but again, it would depend on a currently non-existent distribution chain.

It is necessary to improve monitoring throughout the supply chains to increase compliance with management measures (e.g. satellite monitoring of SMS vessels; monitoring of diseases, discards, and juveniles). To improve the quality of fish (especially *anchoveta*) landed for DHC, it would be advisable to increase the awareness of fishermen and the personnel who inspect landing points about food-safety issues to reduce in-plant discards.

Policy measures should also be adopted to improve production of *anchoveta* DHC products, such as establishing a quota system for SMS fleets and/or allowing all fleets to land fish for either DHC or IHC as long as minimum requirements (e.g. fish preservation) for each are fulfilled [32]. These measures would also help reduce in-plant discards, rationalise pricing for raw *anchoveta* and reduce IUU.

### Scenarios 1 and 2: *Anchoveta* for reduction or for food?

S1 represents the status quo, that is to say the management strategy of the reference year (2011) retained after the beginning of the simulation (2004), and extrapolated into the future. This scenario is sustainable from the perspective of managing *anchoveta* stock but sub-optimal in socio-economic aspects. Indeed, the current redistribution of the fish processing industry profit is limited, due to several factors [32], and does not provide enough income to the lowest economic classes of the Peruvian population to alleviate their hunger and nutritional issues. S2 would improve sustainability in a variety of ways. For instance, by extracting nearly the same amount of biomass without reducing the mean TL of landings or the proportion of predatory fish in the ecosystem (Figure 6), gross profit would increase by a factor of 1.2 due to increased activity of DHC processing industries. Similarly, employment would increase by a factor of 1.5 and available protein for consumers by a factor of 2.5. The environmental costs of these improvements represent a 1.4-fold increase compared to S1. The implications of S2 are complex: for instance, gross profit would be generated by more firms than at present, and national distribution chains would have to be developed. Moreover, because it is unlikely that Peruvian consumers will consume all the additional *anchoveta* production (which increases by a factor of 2.8 in whole-fish equivalents), an export market must be found, which remains uncertain. But if demand for exported canned Peruvian *anchoveta* became dominant, prices in the domestic market could increase [32]. Nonetheless, S2 would be more sustainable at the national level than S1, especially if profit becomes more evenly distributed within the DHC sector.

### Scenario 3: *Anchoveta* today or hake tomorrow?

The goal of S3 is tempting: allow over- or fully-exploited stocks to increase to the point that they can be exploited again (hopefully more sustainably than in the past). Decreasing *anchoveta* fishing mortality to 50% over at least 10 years would increase other NHCS stocks, notably hake (by 18% in biomass). An associated increase in hake catches (by a factor of ∼1.4) would thus be possible, and a similar increase is predicted for stocks of other predators –e.g. conger (*Ophichthus remiger*), flatfish, horse mackerel (*Trachurus murphyi*), pinnipeds and seabirds–, whereas a decrease of a few species that compete with *anchoveta* (e.g. other small pelagic species) and of cetaceans is predicted. The implications of such a dramatic change in resource exploitation are diverse but underestimated due to considering only species biomass: total biomass, biotic resource use, total gross profit, employment and environmental impacts would all decrease (by factors of 0.4, 0.3, 0.3, 0.2 and 0.4, respectively). Moreover, the mean TL of landings slightly increases due to the change in the proportions of *anchoveta* and hake landed. The proportion of predators (fish and others) in the ecosystem slightly decreases (from 0.19 to 0.18) in this scenario because biomass of *anchoveta* increases slightly more than that of predators. The inverse fishing pressure increases due to the drastic reduction in total landings (Figure 9). The available amount of protein of *anchoveta* and hake for Peruvian consumers would also decrease (by a factor of 0.3), but is likely to be partly compensated (if not overcompensated) by an increase in landings of other species caught for DHC (Figure 8; Figure C2 in File S1).

Overall, according to these indicators, S3 seems less preferable than S1 and S2 despite some ecological and environmental improvements. Moreover, obtaining the national consensus required to decrease exploitation of the *anchoveta* stock so dramatically would be a daunting endeavour, to say the least. Nonetheless, this scenario deserves more in-depth study, varying the exploitation rates less drastically and taking into account all species in the ecosystem that are exploited or potentially exploitable by fisheries or for tourism. Furthermore, how changes in volumes of fish landings could affect fishing costs and prices of each species should be considered in scenarios. Expected changes in fishing costs were already taken into account through our CPUE-adjusted fuel use intensities. Only minor additional changes can be expected because the major fisheries are already large and mature industrial ones, preventing major changes due to economy of scale. In contrast, the existing increasing trend in fishmeal prices may be exacerbated by a decrease in Peruvian catches of *anchoveta* in S3. Indeed, the Peruvian share of this commodity is over 40%, its global production is decreasing and its sustained demand is price-inelastic [32], [120]. However, the concomitant increase in Peruvian hake production (by a factor of 1.4) will not result in lower hake prices because the Peruvian share of this commodity is too low (3–5% [45]) to impact its global market. As a result, the decrease in gross profit of the Peruvian fishing industry should be lower than predicted in S3.

## Conclusions

The proposed framework, as illustrated with the Peruvian case study, provides a multi-criteria toolset for decision-making to improve fishfood supply chain dynamics. Scenario analysis confirmed previous speculations that an increase in the proportion of *anchoveta* destined for DHC would positively contribute to Peru’s sustainable development (S2). It also indicated that a dramatic reduction in *anchoveta* landings would not be, in general, positive for the country (S3), although this scenario deserves deeper investigation (e.g. consideration of all species, sensitivity analysis of more realistic changes in exploitation rates to estimate optimal levels). The preservation of ecosystem services should also be considered in more detail.

Due to the huge size of the reduction industry and its supplier fisheries, results per functional unit do not align with absolute results per industry (DHC vs. IHC vs. aquaculture). Indeed, in absolute terms, the activities in Peru with most impact at present are those related to capturing and reducing *anchoveta* into fishmeal and fish oil. As a result, the best opportunities for improving the environmental and socio-economic performance of Peruvian *anchoveta* supply chains would be related to management and policy changes that improve the sustainability of the reduction industry and its suppliers.

## Supporting Information

File S1 Supplementary Material: tables, figures, and references.
(DOC)
