# Supplementary material for: Coupled Ecosystem/Supply Chain Modelling of Fish Products from Sea to Shelf: The Peruvian Anchoveta Case
Source: PLoS One. 2014 Jul 8;9(7):e102057. doi: 10.1371/journal.pone.0102057 (PMC4086974; doi:10.1371/journal.pone.0102057)
Supplement: File S1 — Supplementary Material: tables, figures, and references. (DOC) [file pone.0102057.s001.doc]

# Coupled ecosystem/supply chain modelling of fish products from sea to shelf: the Peruvian *anchoveta* case

Angel Avadí ^1,2^,*, Pierre Fréon ^2^, Jorge Tam ^3^

^1^ Université Montpellier 2 – Sciences et Techniques, Montpellier, France.

^2^ Institut de Recherche pour le Développement (IRD), UMR212 EME IFREMER/IRD/UM2, Sète, France.

^3^ Instituto del Mar del Perú (IMARPE), Callao, Peru.

* Email: [angel.avadi@ird.fr](mailto:angel.avadi@ird.fr)

# Supplementary Material: tables, figures, and references

## A. Classification of modelling tools and justification of retained models

Various types of models linking the natural and socio-economic systems can be clustered into the following categories: ecological/ecosystem, bio-economic and social-ecological systems (SES) modelling [1,2]. In general terms, ecological models attempt to explain the effects of harvesting resources on the providing ecosystem (including interactions between species) while bio-economic models analyse those interactions in both directions. The emerging cross-cutting field of SES extends bio-economic models by including non-linear behaviour and by treating links from the ecosystems as ecosystem services rather than as utility-providing resources. Such complexity is possible due to the fact that SES profit from a variety of modelling fields, and have been applied to a variety of applications: fisheries, rangeland, wildlife, bio-economics, ecological economics, resilience, and complex systems [2].

Several typologies exist, but in general marine ecosystem models can be classified into the following categories [3]: whole ecosystem models, dynamic multi-species models (Minimum Realistic Models, MRM), and dynamic system models —including Individual-Based Models, IBM, such as OSMOSE [4], as well as biogeochemical models such as ATLANTIS [5]—.

An emerging topic in marine ecosystem modelling is the concept of end-to-end ecosystem models. The end-to-end modelling framework attempts to include the effect of both climate change (through the higher trophic levels) and anthropogenic intervention in multi-trophic models [6,7]. Those models arise out of the needs of ecosystem-based management, which demands models considering climate change and time and space variations, such as OSMOSE and EwE/ECOSPACE [6]. A key research topic in end-to-end modelling is the type of coupling between hydrodynamic, low and high trophic levels sub-models: one-way forcing/linking/coupling or two-way coupling [6,8,9]. The latter allows for dynamic feedbacks related to density-dependent responses of high trophic level organisms and to interaction between biological and physical processes [6]. Moreover, feedbacks add mathematical and computational complexity to the model.

A review of applications suggests supply chain redesigning, validation and verification, sensitivity analysis, optimisation, robustness, risk and uncertainty analysis, etc; are amongst the issues addressed by supply chain modelling [10]. Various approaches to supply chain modelling have been described and several typologies proposed [11–19]. Regarding the overall approach (meta-model, framework) required to guide supply chain modelling, more than one has been proposed, but the Supply Chain Operations Reference (SCOR), a descriptive type, provides a widely accepted way of depicting supply chains in a standardised fashion that allows for model comparison [18,20]. SCOR is one of the most widely used frameworks in business and research [21]. Further guidelines have been described [15,18], and a number of methods to assess supply chain performance have been contrasted [22].

SCM applied to food supply chains addresses issues such as food safety and risk management [23], redesigning the supply chain towards performance improvements [24], trade-offs between logistic costs and final product quality [25,26], accounting and reducing food waste [27], etc.

Fishfood supply chains face specific supply chain challenges, such as: quality variation between batches, given that most wild caught species are identified by batches; variation and uncertainty of catches leading to complex trading systems such as auction markets [26]; sustainability issues such as trade-offs between resource base conservation and socio-economic objectives [28]; traceability [29]; shelf life and safety; subsidies and rights; etc.

The field of futures research has produced several scenario typologies. The typology proposed by [30] seems particularly suitable for scenario building in association to socio-economic modelling, given its organisation around key questions about the future a model/scenario might attempt to answer: “What will happen?”, “What can happen?” and “How can a specific target be reached?”.

## B: Supporting tables and figures

Table B1. Chronology of key fisheries legislation in Peru.

| **Year** | **Legal instrument** | **Issue** |
| --- | --- | --- |
| 1961 | Law No. 13825 | Apply 14% tax on fishmeal exports |
| 1962 | Decree Law 14195 | Regulating instalment of fishmeal factories |
|  | Decree Law 14228 | Fishmeal exports allowed only through cooperatives |
| 1963 | Supreme Decree 16-63-PE | Setting up the National Fisheries Council |
|  | Supreme Decree 18-63-PE | Establishing exporting quotas and a new licenses’ system for fishmeal processing plants |
|  | Supreme Decree 77-63-PE | Officially recognizing the Peruvian Fishmeal Consortium |
| 1964 | Law No. 15048 | New tax system for fishmeal exports, valid for 10 years (0.22 USD per tonne fishmeal and 0.11 USD per tonne fish oil) |
|  | Supreme Decree 07-64-PE | Establishment of Peruvian Marine Research Institute |
| 1965 | Supreme Decree 05-65-PE | First *anchoveta* closed season |
| 1967 | Law No. 16694 | Law for Fisheries Promotion |
| 1968 | Law No. 17403 | Setting import free taxes for fishmeal equipment |
| 1969 | Decree Law 180261 | Establishment of Ministry of Fisheries |
| 1970 | Decree Law 18196 | Establishment of Fisheries Development Fund |
|  | Decree Law 18253 | Establishment of State Company for commercialization of fishmeal and fish oil |
| 1971 | Decree Law 18810 | (First) General Fisheries Act (repealed) |
| 1988 | Law No. 24790 | (Second) General Fisheries Act (repealed) |
| 1992 | Decree Law 25977 | (Third) General Fisheries Act (featuring prohibition of fleet enlargement and increasing fishmeal plant capacity) |
| 1994 | Supreme Decree 01-94-PE | Regulation for the Fisheries Act (repealed) |
| 1997 | Supreme Decree 001-97-PE | Publication of official list of fishing vessels |
|  | Supreme Decree 781-97-PE | Declaration of *anchoveta* and sardine as fully-exploited species |
| 1998 | Law No. 26920 | Law regulating wooden fleet vessels with holding capacity 32.6-110 m^3^ (Vikingas, operating illegally before the law). It excludes Vikingas operators from the fleet enlargement limitations of Decree Law 25977 |
| 2001 | Supreme Decree 012-2001-PE | Regulation for the Fisheries Act, defines artisanal (SMS) vessels as those featuring a holding capacity of up to 32.6 m^3^ and 15 m length |
|  | Supreme Decree 040-2001-PE | Sanitary standard for fisheries and aquaculture resources |
| 2002 | Supreme Decree 001-2002-PE | Establishes catches of sardine, jack mackerel and chub mackerel can be only dedicated to DHC |
|  | Supreme Decree 007-2002-PE | Fishing permits and vessel decommissioning |
| 2003 | Supreme Decree 026-2003-PE | Regulation for the Vessel Monitoring System |
|  | Supreme Decree 027-2003-PE | Establishment of the Surveillance and Control Program for Fisheries and Landings |
| 2005 | Ministerial Resolution 043-2005-PRODUCE | Establishes rules for processing of fish residues from small- and medium-scale landing ports |
| 2006 | Supreme Decree 024-2006-PRODUCE | Establishment of “fishing rights” for landings destined for reduction (0.25% of the average monthly FOB value of 1 t of fishmeal, per landed t of *anchoveta*) |
|  | Ministerial Resolution 205-2006-PRODUCE | Establishes rules for processing of fish residues and discards from processing for direct human consumption |
| 2007 | Supreme Decree 002-2007-PRODUCE | Declaration of direct human consumption of *anchoveta* and jumbo squid to be of strategic and national interest |
| 2008 | Legislative Decree 1084 | Introduction of individual vessel quotas (to end the race for fish under the total allowable quota). Imposition of a USD 1.95 fee per landed t |
|  | Supreme Decree 021-2008-PRODUCE | Regulation for Legislative Decree 1084 |
| 2009 | Supreme Decree 009-2009-PRODUCE | Establishes the regulations for an individual vessel quota system in the South Zone of Peru |
| 2010 | Supreme Decree 010-2010-PRODUCE | Regulation for Fisheries Management of *Anchoveta* for Direct Human Consumption (DHC) |
|  | Supreme Decree 018-2010-PRODUCE | Prohibition of building new vessels over 5 m^3^ of holding capacity |
|  | Supreme Resolution 028-2010-PRODUCE | Creation of the National Council for the Promotion of the Resources *anchoveta* and *pota* (flying giant squid, *Dosidicus gigas*) |
| 2011 | Supreme Decree 005-2011-PRODUCE | Regulation for processing of fish residues and discards |
|  | Supreme Decree 017-2011-PRODUCE | Modifies the regulation for processing of fish residues and discards |
| 2012 | Supreme Decree 005-2012-PRODUCE | Subdivision of the direct human consumption fleet into small-scale (<10 m^3^) and medium-scale (10-32.6 m^3^). Also assigns exclusive fishing rights within the first 5 nautical miles to the former and from 5 to 10 nautical miles to the latter. |
|  | Ministerial Resolution 433-2012-PRODUCE | Complementary regulation establishing that small- and medium-scale vessels landing *anchoveta* for DHC must have a purchase agreement with fish processing plants. |
|  | Supreme Decree 008-2012-PRODUCE | Establishment of the obligation to report fishing grounds where juveniles are present. |
| 2013 | Judgement by the Supreme Court (November) | The Supreme Court declared unconstitutional the exclusivity of the 5 to 10 nautical miles for the medium-scale fleet as it appears in Supreme Decree 005-2012. Currently under appeal by PRODUCE. |

Based on [31–34].

**Table B2.** Detailed mass balances, gross profit and employment figures of the modelled scenarios (economy-wide), excluding distribution.

| **Products** | | ***Status quo* (2011)** | | | **Scenario 1 (2021)** | | | **Scenario 2 (2021)** | | | **Scenario 3 (2021)** | | |
| --- | --- | --- | --- | --- | --- | --- | --- | --- | --- | --- | --- | --- | --- |
|  |  | **Biomass (kt)** | **Gross profit**  **(1000 USD)** | **Employment (direct jobs)** | **Biomass (kt)** | **Gross profit**  **(1000 USD)** | **Employment (direct jobs)** | **Biomass (kt)** | **Gross profit**  **(1000 USD)** | **Employment (direct jobs)** | **Biomass (kt)** | **Gross profit**  **(1000 USD)** | **Employment (direct jobs)** |
| *Anchoveta* | Landings IHC | 6 996 | 233,664 | 14 903 | 6 574 | 219,546 | 14 002 | 6 139 | 205,037 | 13 077 | 3 958 | 132,195 | 8 431 |
|  | Discards | 277.1 | 9,255 | N/A | 276.8 | 9,246 | N/A | 276.8 | 9,246 | N/A | 166.7 | 5,567 | N/A |
|  | FM | 1635 | 233,922 | 15 705 | 1561 | 223,437 | 14 960 | 1458 | 208,670 | 13 971 | 940.2 | 134,538 | 9 008 |
|  | FO | 328.5 | 47,003 |  | 308.6 | 44,163 |  | 288.2 | 41,244 |  | 185.8 | 26,592 |  |
|  | Landings DHC | 109.0 | 8,078 | 2 280 | 247.7 | 18,353 | 5 181 | 682.1 | 50,545 | 14 270 | 149.1 | 11,051 | 3 120 |
|  | Canning | 84.2 | 28,091 | 653 | 191.3 | 63,825 | 1484 | 526.8 | 175,774 | 4 088 | 115.2 | 38,431 | 894 |
|  | Freezing/fresh | 14.7 | 9,099 | 1443 | 33.5 | 20,673 | 3 278 | 92.1 | 56,934 | 9 026 | 20.1 | 12,448 | 1974 |
|  | Curing/salting | 10.1 | 7,660 | 848 | 22.9 | 17,404 | 1 927 | 63.1 | 47,932 | 5 306 | 13.8 | 10,480 | 1160 |
| Hake | Landings | 31.4 | 28,628 | 5 096 | 46.3 | 42,208 | 7 513 | 46.3 | 42,208 | 7 513 | 84.9 | 77,420 | 13 781 |
|  | Discards | 4.7 | 4,294 | N/A | 8.2 | 7,448 | N/A | 8.2 | 7,448 | N/A | 15.0 | 13,662 | N/A |
| Aquaculture | Trout | 20.0 | 6,666 | 829 | 29.4 | 9,809 | 1 220 | 29.4 | 9,809 | 1 220 | 29.4 | 9,809 | 1220 |
|  | Tilapia | 2.4 | 4,387 | 2 114 | 4.0 | 7,275 | 3 504 | 4.0 | 7,275 | 3 504 | 4.0 | 7,275 | 3 504 |
|  | Black pacu | 0.7 | 858 | 675 | 1.4 | 1,852 | 1 457 | 1.4 | 1,852 | 1457 | 1.4 | 1,852 | 1457 |
| **Totals** | | **9 232** | **608 065** | **44 546** | **9 020** | **668 545** | **54 527** | **9 331** | **847 279** | **73 433** | **5 502** | **462 090** | **44 549** |

Notes. Gross profit = Revenues – Production costs. IHC: Indirect Human Consumption, DHC: Direct Human Consumption, FM: Fishmeal, FO: Fish oil.

**Table B3. Detailed environmental, nutritional, and energy efficiency scores of the modelled scenarios (economy-wide), excluding distribution.**

| **Products** | | ***Status quo* (2011)** | | | **Scenario 1 (2021)** | | | **Scenario 2 (2021)** | | | **Scenario 3 (2021)** | | |
| --- | --- | --- | --- | --- | --- | --- | --- | --- | --- | --- | --- | --- | --- |
|  |  | **ReCiPe single score (Pt)** | **BRU**  **(kt C∙kt^-1^)** | **Available protein for DHC (t)** | **ReCiPe single score (Pt)** | **BRU**  **(kt C∙kt^-1^)** | **Available protein for DHC (t)** | **ReCiPe single score (Pt)** | **BRU**  **(kt C∙kt^-1^)** | **Available protein for DHC (t)** | **ReCiPe single score (Pt)** | **BRU**  **(kt C∙kt^-1^)** | **Available protein for DHC (t)** |
| *Anchoveta* | Landings IHC | 98 660 | 38 960 | N/A | 92 699 | 36 606 | N/A | 86 573 | 34 187 | N/A | 55 817 | 22 042 | N/A |
|  | Discards | 3 908 | 60.2 | 810.6 | 3 904 | 60.1 | 1842 | 3 904 | 60.1 | 5 072 | 2 351 | 36.2 | 1109 |
|  | FM | 186 522 | 42 738 | N/A | 178 161 | 40 822 | N/A | 166 387 | 38 124 | N/A | 107 276 | 24 580 | N/A |
|  | FO | 373 269 | 43 447 | N/A | 350 716 | 40 822 | N/A | 327 538 | 38 124 | N/A | 211 177 | 24 580 | N/A |
|  | Landings DHC | 3 336 | 607.0 | 20 785 | 7 580 | 1379 | 47 225 | 20 875 | 3 799 | 130 057 | 4 564 | 830.5 | 28 435 |
|  | Canning | 72 922 | 768.9 | 17 891 | 165 687 | 1747 | 40 651 | 456 304 | 4 812 | 111 953 | 99 765 | 1052 | 24 477 |
|  | Freezing/fresh | 554.8 | 109.3 | 2 807 | 1261 | 248.4 | 6 379 | 3 472 | 684.1 | 17 567 | 759.1 | 149.6 | 3 841 |
|  | Curing/salting | 11 349 | 289.2 | 1853 | 25 787 | 657.2 | 4 210 | 71 017 | 1810 | 11 594 | 15 527 | 395.7 | 2 535 |
| Hake | Landings | 3 473 | 6 957 | 5 220 | 5 121 | 10 257 | 7 696 | 5 121 | 10 257 | 7 696 | 9 393 | 18 815 | 14 116 |
|  | Discards | 521.0 | 144.0 | 783.0 | 919.7 | 249.8 | 1358 | 903.6 | 249.8 | 1358 | 1658 | 458.2 | 2 491 |
| Aquaculture | Trout | 16 940 | 998.9 | 3 669 | 24 926 | 1470 | 5 399 | 24 926 | 1470 | 5 399 | 24 926 | 1470 | 5 399 |
|  | Tilapia | 3 812 | 42.5 | 444.0 | 6 320 | 70.5 | 736.2 | 6 320 | 70.5 | 736.2 | 6 320 | 70.5 | 736.2 |
|  | Black pacu | 694.8 | 9.7 | 99.7 | 1499 | 20.9 | 215.1 | 1499 | 20.9 | 215.1 | 1499 | 20.9 | 215.1 |
| **Totals** | | **670 491** | **135 132** | **54 362** | **773 498** | **134 410** | **115 710** | **1 062 269** | **133 668** | **291 647** | **471 257** | **94 500** | **83 354** |

Notes. IHC: Indirect Human Consumption, DHC: Direct Human Consumption, FM: Fishmeal, FO: Fish oil.

**
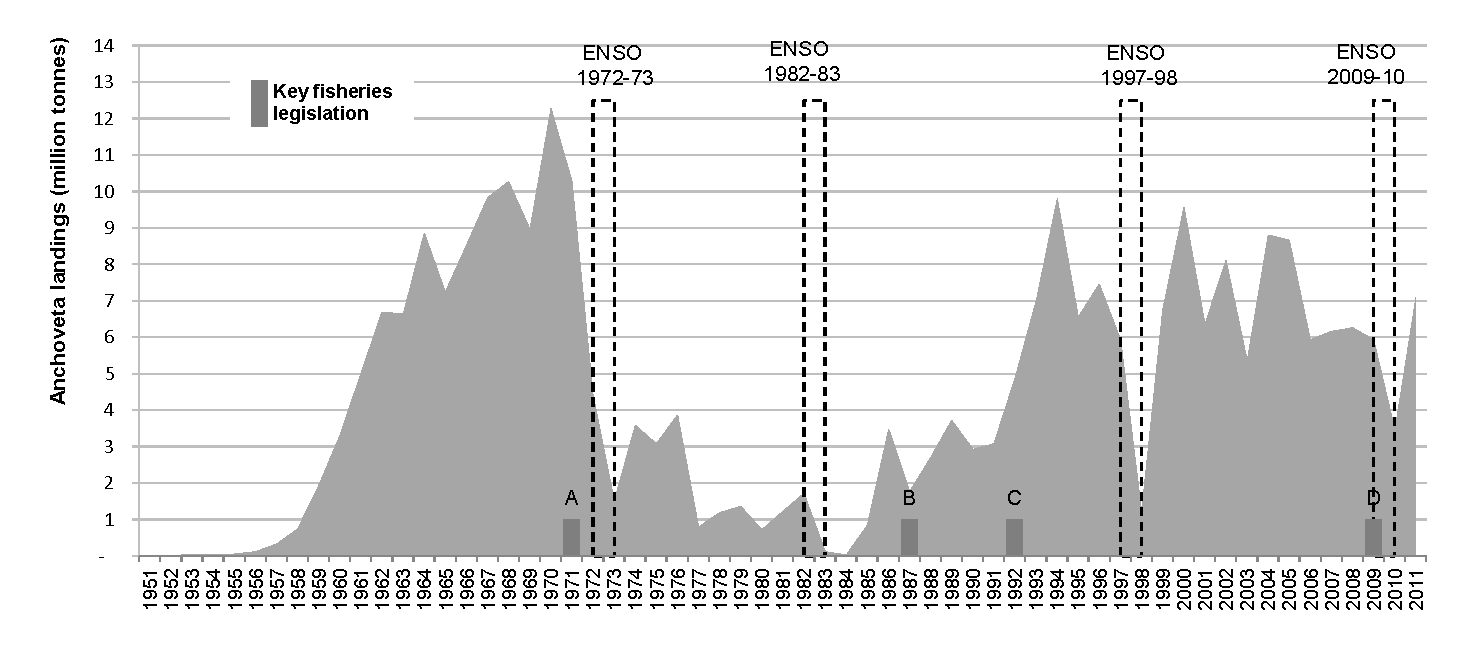
**

**Figure B1.** Historical annual *anchoveta* landings and critical El Niño (ENSO) and policy events (1951-2011). A - (First) General Fisheries Act, B - (Second) General Fisheries Act, C - (Third) General Fisheries Act, D - Legislative Decree 1084 (individual vessel quota system). Source: statistics from FishStatJ and PRODUCE. Inspired from Figure 2 in [31].


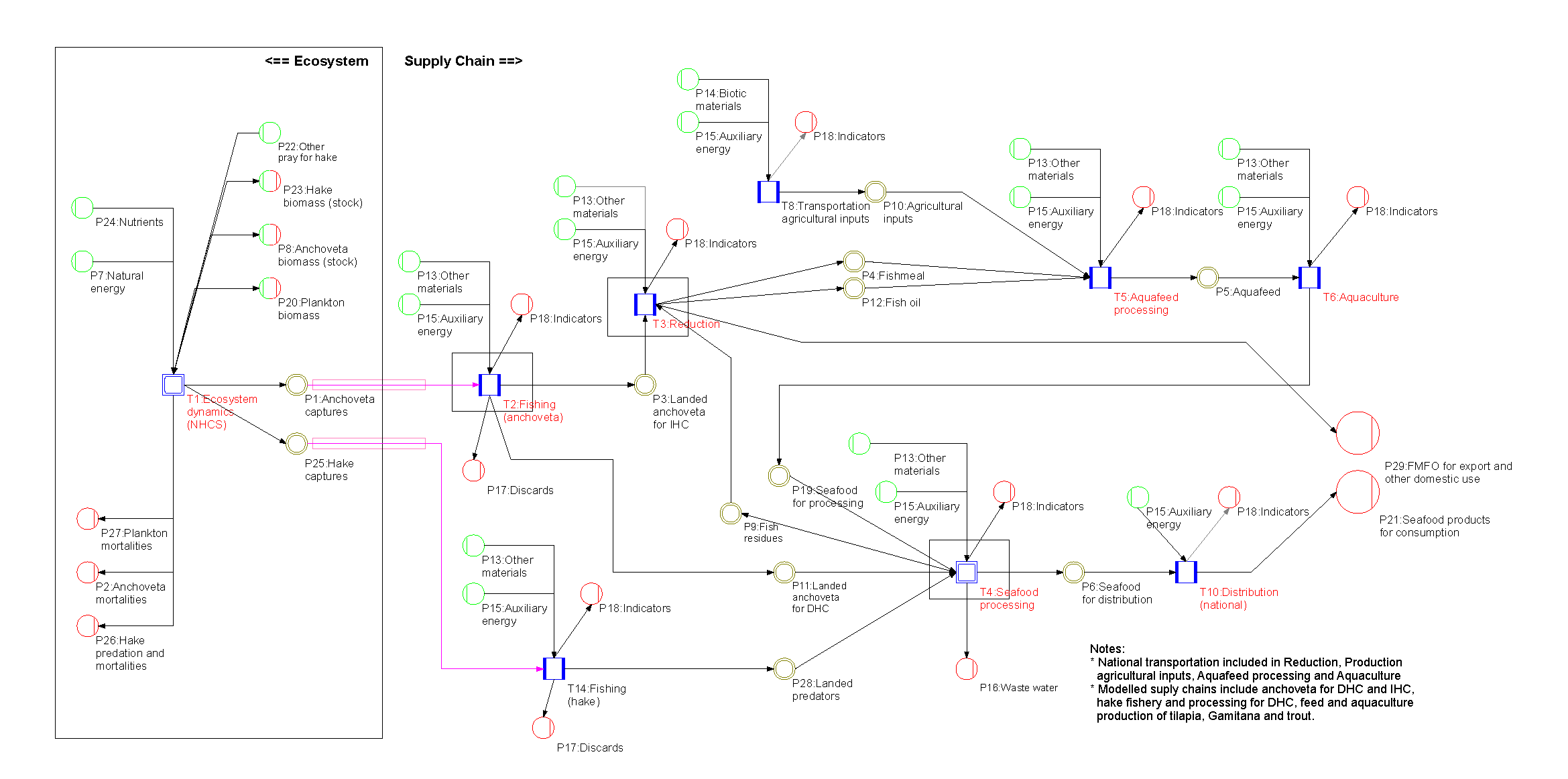


**Figure B2.** Material flow model of the Peruvian *anchoveta* supply chains, featuring interactions with the Northern Humboldt Current System (NHCS). The “Supply Chain” part of the model can be used separately to generate mass balances of alternative exploitation/production mixes (every arrow can be modified manually and the rest of the system would be recalculated for such intervention).

**
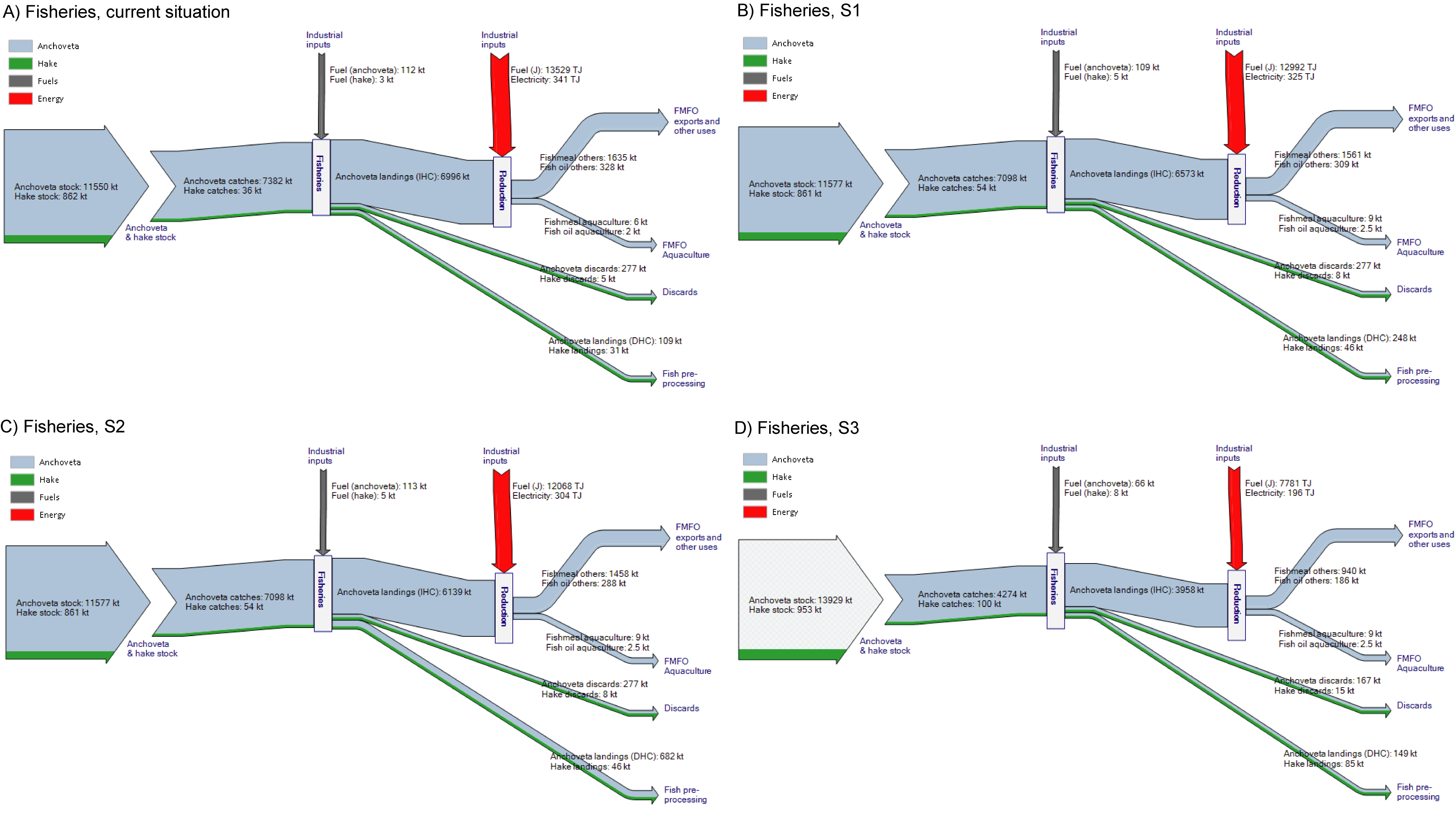
**

**Figure B3.** Mass and energy Sankey diagrams for fisheries across scenarios.

**
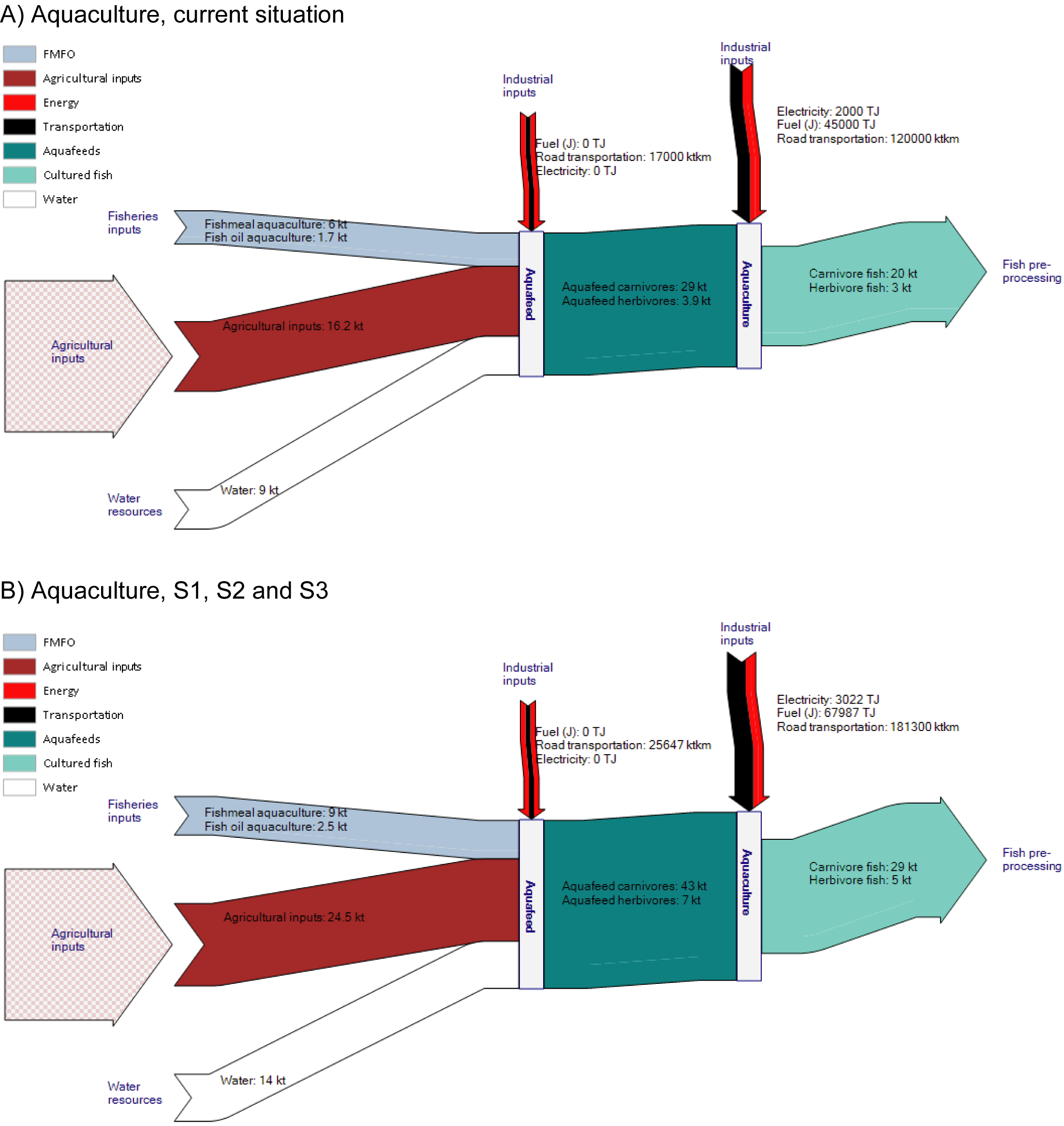
**

**Figure B4.** Mass and energy Sankey diagrams for aquaculture across scenarios.

**
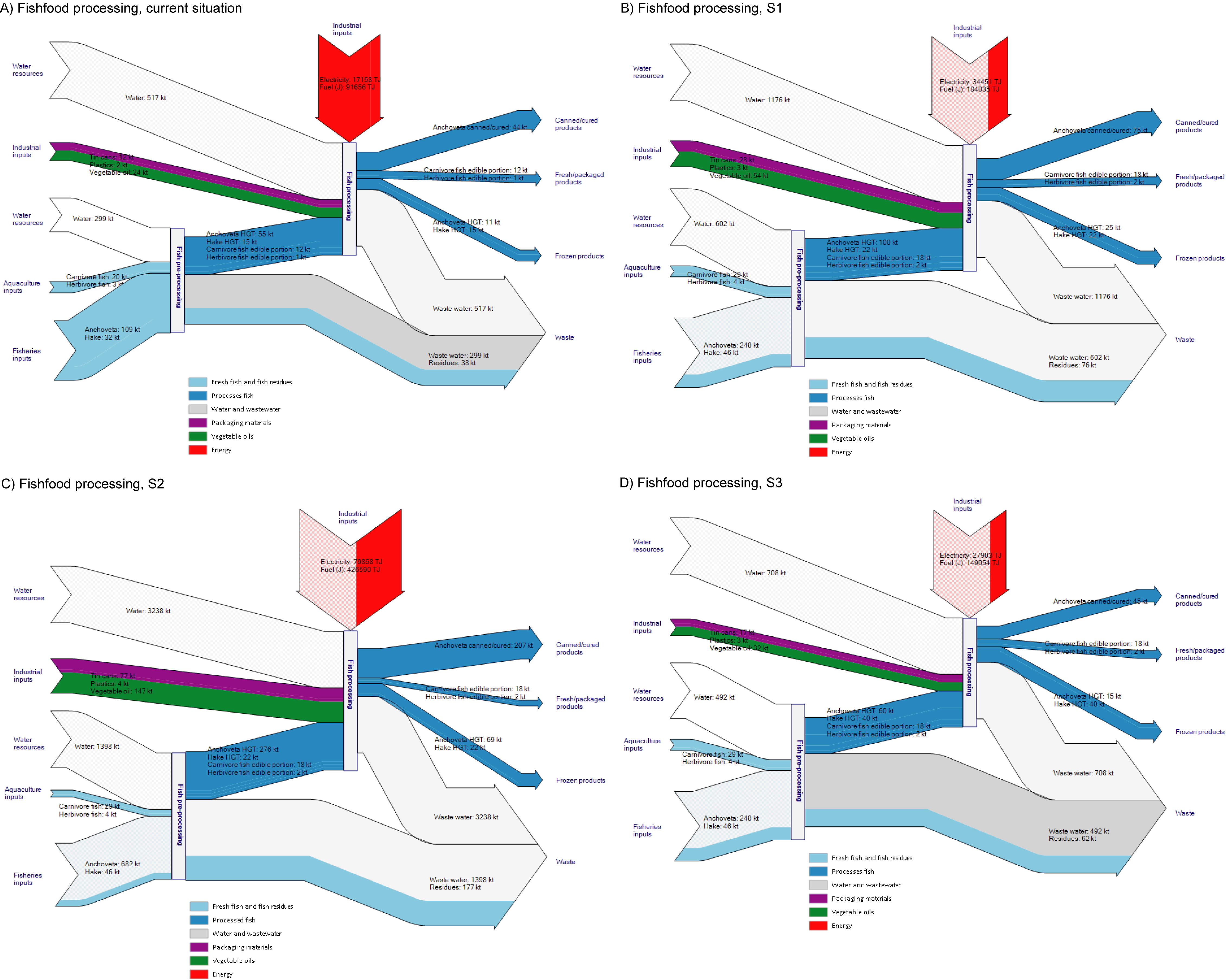
**

**Figure B5.** Mass and energy Sankey diagrams for fishfood processing across scenarios.


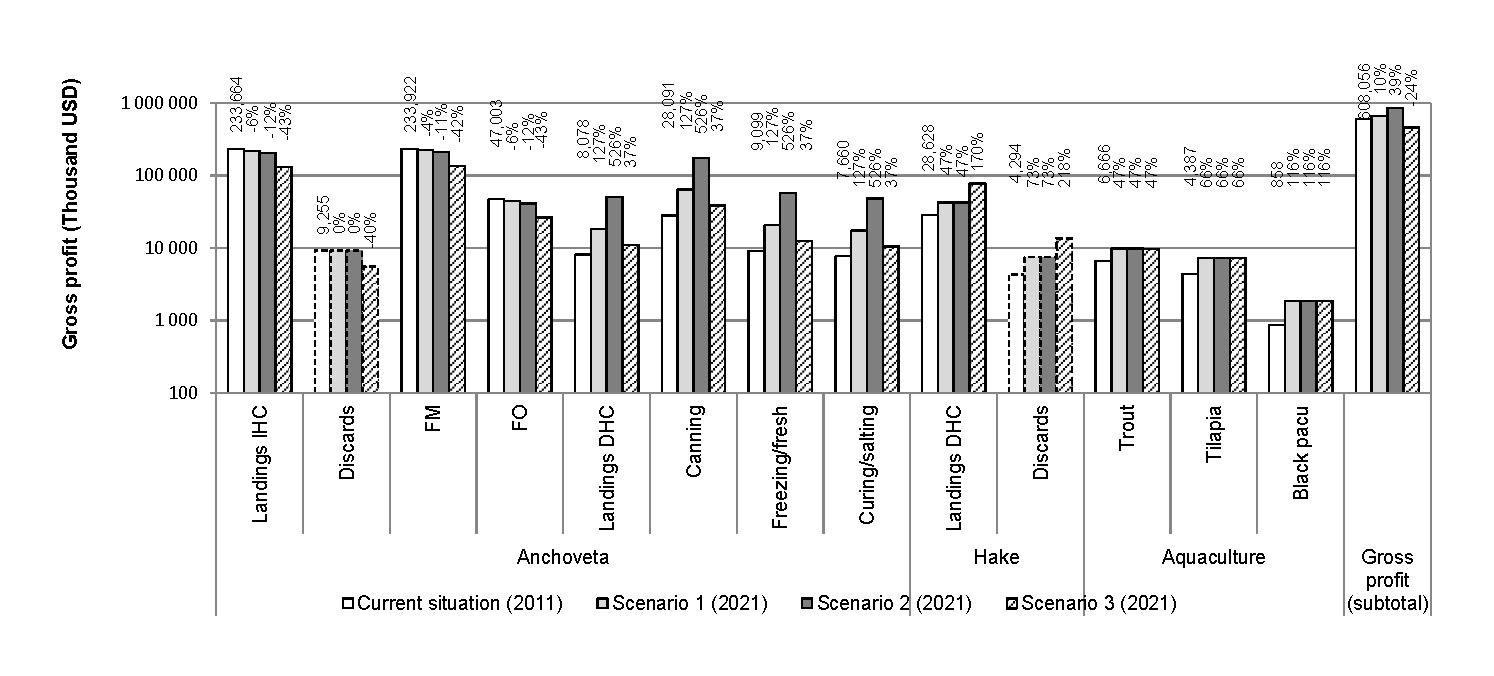


Figure B6. Economic outputs associated to the alternative exploitation scenarios. Per key product, on a log_10_ scale. Percentages represent variation from the current situation. Gross profit = revenues – production costs.


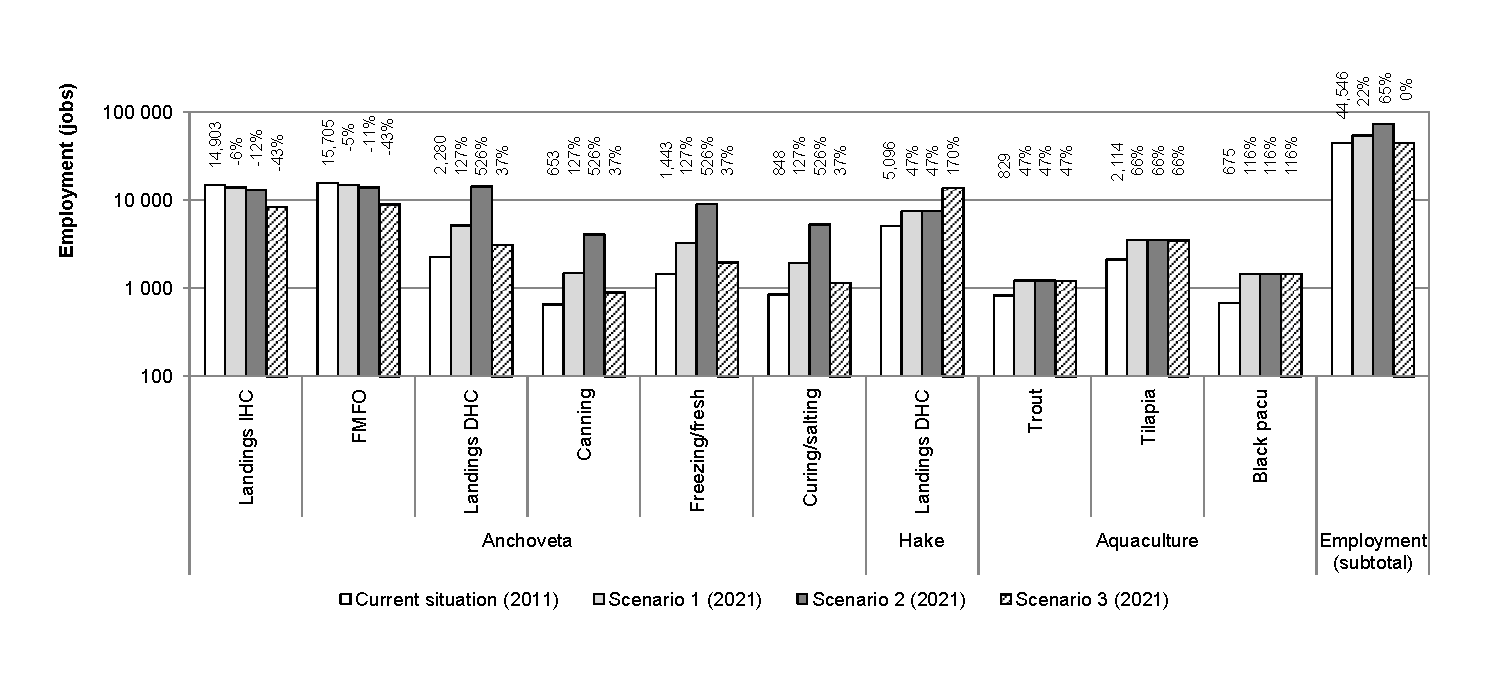


Figure B7. Employment associated to the alternative exploitation scenarios. Per key product, on a log_10_ scale. Percentages represent variation from the current situation.


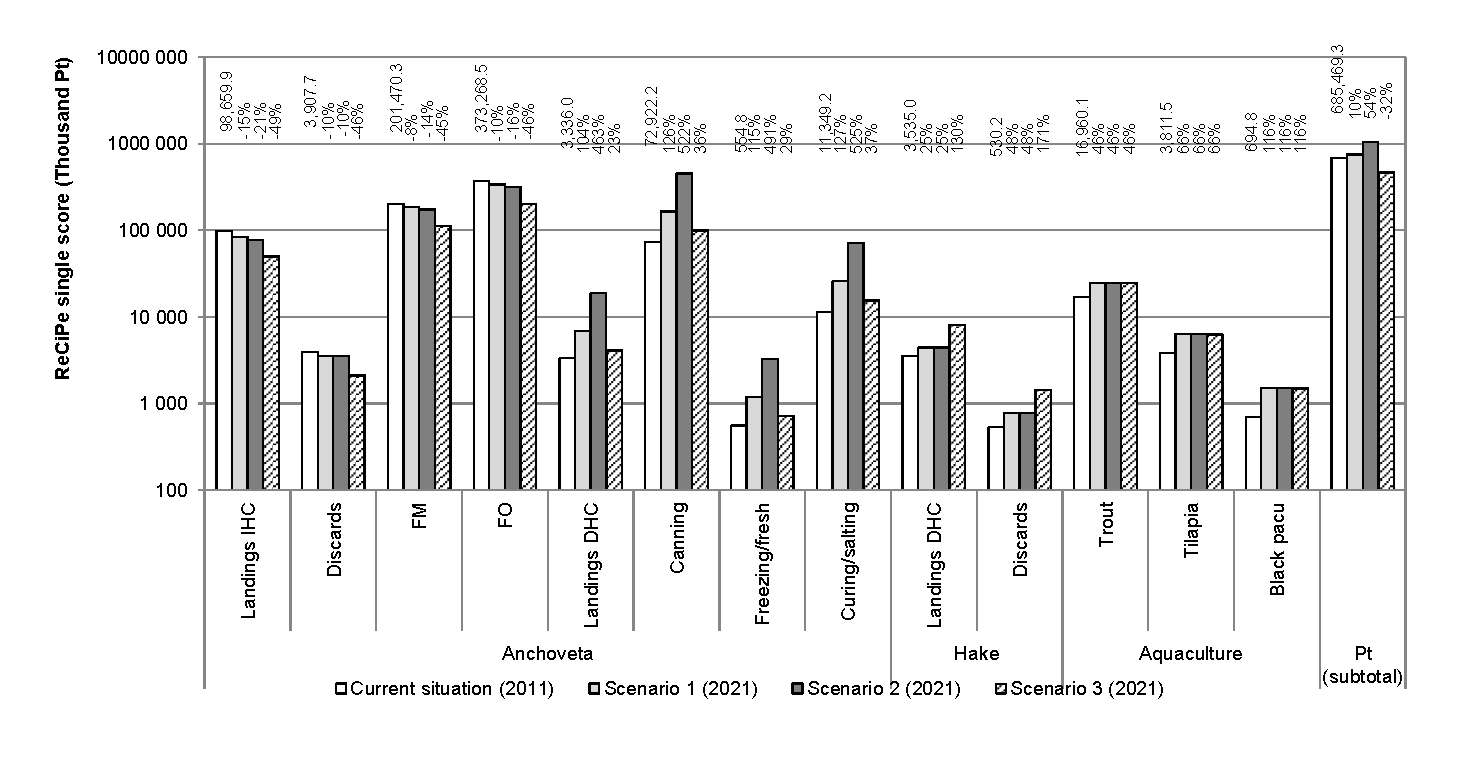


Figure B8. Environmental score (ReCiPe single score) associated to the alternative exploitation scenarios. Per key product, on a log_10_ scale. Percentages represent variation from the current situation.


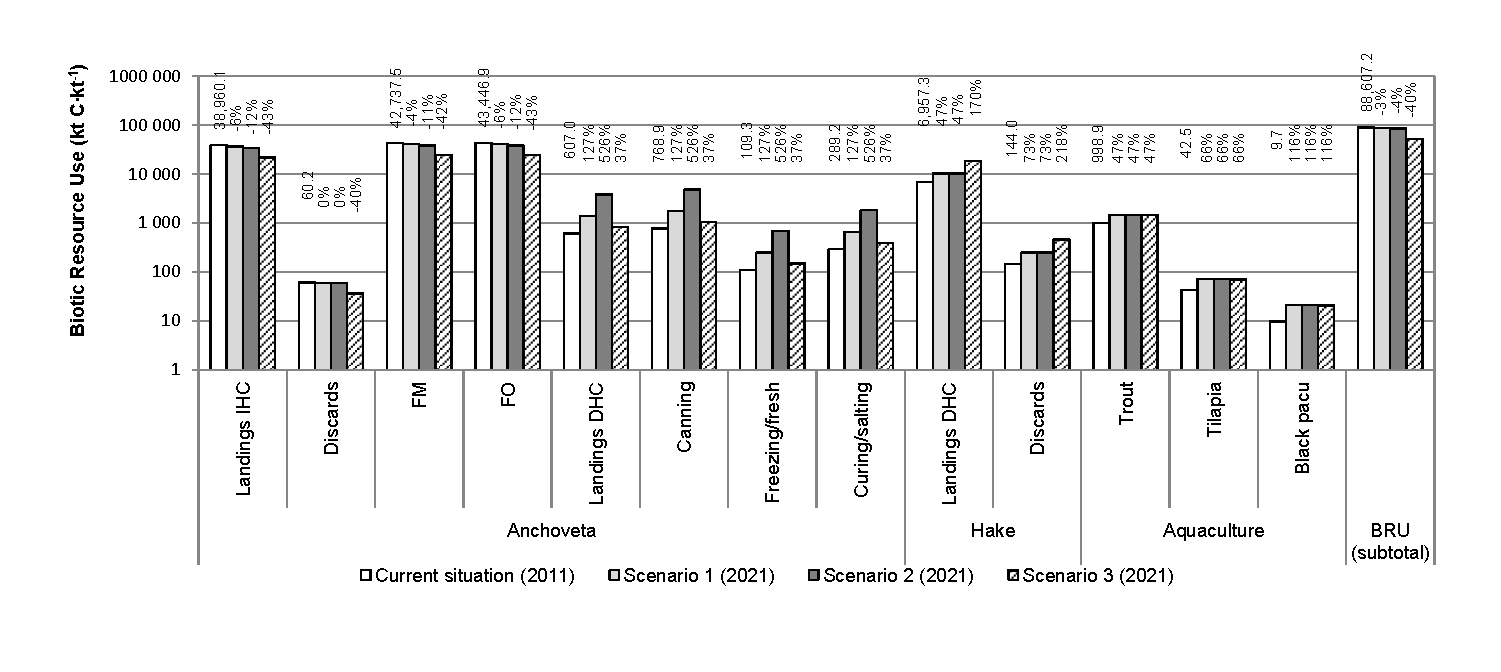


Figure B9. Biotic Resource Use associated to the alternative exploitation scenarios. Per key product, on a log_10_ scale. Percentages represent variation from the current situation.


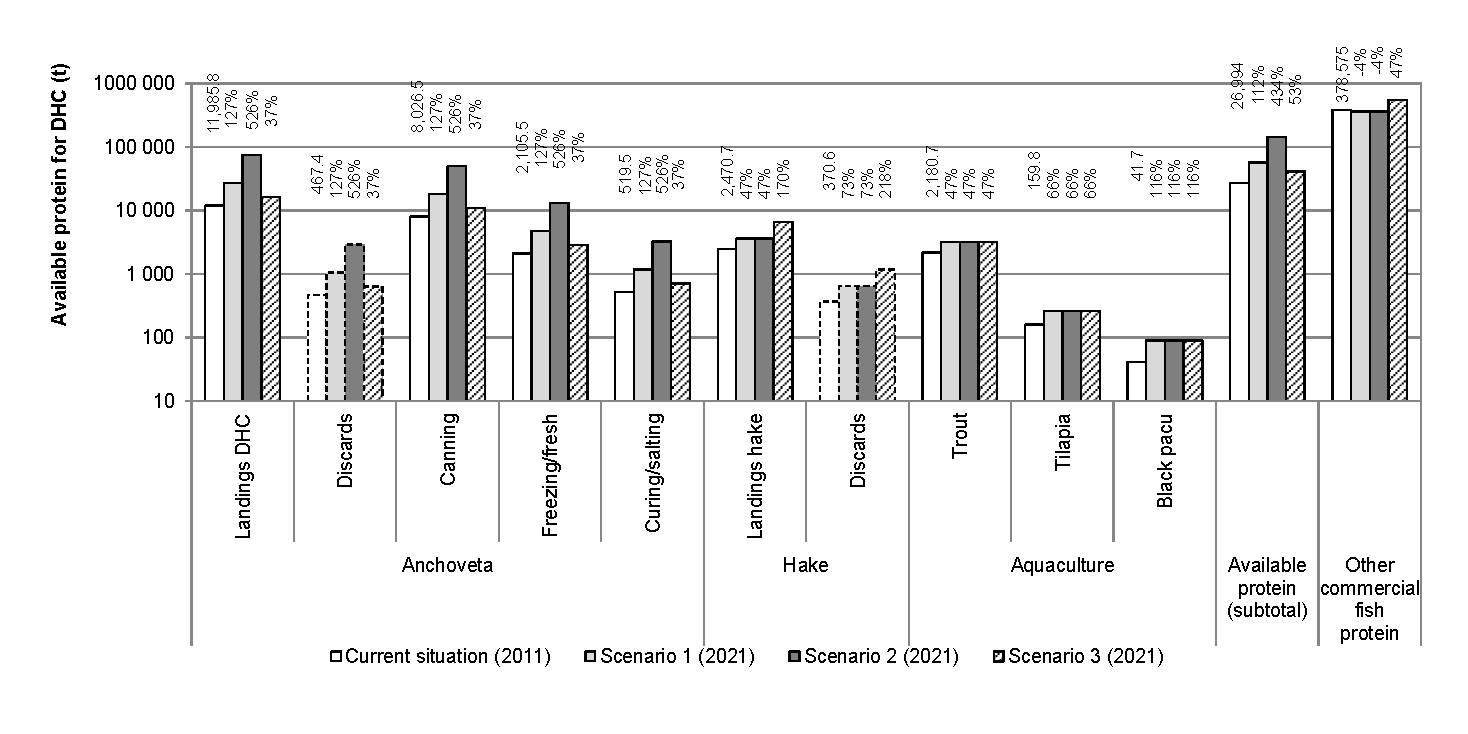


Figure B10. Human nutritional protein (as a proxy of nutritional benefits) delivered by the alternative exploitation scenarios. Per key product, on a log_10_ scale. Percentages represent variation from the current situation. Other commercial fish landed refers to catfish (*Galeichthys peruvianus*), flatfish (*Paralichthys adspersus*) and Eastern Pacific bonito (*Sarda chiliensis chiliensis*).

## C: EwE outputs for simulation scenarios.

**Table C1.** EwE outputs for reference year 2011. Actual *anchoveta* and hake catches, according to PRODUCE statistics, were 7 382 323 and 36 089, respectively.

| **Species** | **Inputs (t)** |  | **Outputs (t)** |
| --- | --- | --- | --- |
| **Plankton** |  |  |  |
| Solar irradiation (MW) | 165,000,000 | Plankton consumption (*anchoveta*) | 239,429,062 |
| Nutrients | 155,000,000 | Plankton mortalities | 3,657,878,172 |
| Plankton biomass | 23,049,347 | Plankton biomass | 23,049,347 |
| ***Anchoveta*** |  |  |  |
| Plankton consumption | 239,429,062 | *Anchoveta* consumption (hake) | 1,706,090 |
|  |  | *Anchoveta* respiration | 151,182,174 |
|  |  | *Anchoveta* mortalities | 14,335,670 |
|  |  | *Anchoveta* catches | 7,081,409 |
|  |  | *Anchoveta* un-assimilation | 83,800,171.81 |
| *Anchoveta* biomass | 11,550,170 | *Anchoveta* biomass | 11,550,170 |
| **Hake** |  |  |  |
| *Anchoveta* consumption | 1,706,090 | Hake consumption (predators) | 316,953.69 |
| Other prey consumption | 4,359,887 | Hake respiration | 1,948,507 |
|  |  | Hake un-assimilation | 1,859,288 |
|  |  | Hake mortalities | 538,552 |
|  |  | Hake catches | 54,345 |
| Hake biomass | 861,822 | Hake biomass | 861,822.41 |

**Table C2.** EwE outputs for Scenarios 1 and 2 (2021).

| **Species** | **Inputs (t)** |  | **Outputs (t)** |
| --- | --- | --- | --- |
| **Plankton** |  |  |  |
| Solar irradiation (MW) | 165,000,000 | Plankton consumption (*anchoveta*) | 239,809,340 |
| Nutrients | 155,000,000 | Plankton mortalities | 3,665,525,401 |
| Plankton biomass | 23,045,504 | Plankton biomass | 23,045,504 |
| ***Anchoveta*** |  |  |  |
| Plankton consumption | 239,809,340 | *Anchoveta* consumption (hake) | 1,716,384 |
|  |  | *Anchoveta* respiration | 151,182,174 |
|  |  | *Anchoveta* mortalities | 14,340,117 |
|  |  | *Anchoveta* catches | 7,098,017 |
|  |  | *Anchoveta* un-assimilation | 83,933,268.98 |
| *Anchoveta* biomass | 11,577,259 | *Anchoveta* biomass | 11,577,259 |
| **Hake** |  |  |  |
| *Anchoveta* consumption | 1,716,384 | Hake consumption (predators) | 316,206 |
| Other prey consumption | 4,346,013 | Hake respiration | 1,948,507 |
|  |  | Hake un-assimilation | 1,856,457 |
|  |  | Hake mortalities | 538,721 |
|  |  | Hake catches | 54,433 |
| Hake biomass | 860,534 | Hake biomass | 860,534 |

**Table C3. EwE outputs for Scenario 3 (2021).**

| **Species** | **Inputs (t)** |  | **Outputs (t)** |
| --- | --- | --- | --- |
| **Plankton** |  |  |  |
| Solar irradiation (MW) | 165,000,000 | Plankton consumption (*anchoveta*) | 278,270,010 |
| Nutrients | 155,000,000 | Plankton mortalities | 3,602,995,052 |
| Plankton biomass | 22,437,949 | Plankton biomass | 22,437,949 |
| ***Anchoveta*** |  |  |  |
| Plankton consumption | 278,270,010 | *Anchoveta* consumption (hake) | 2,397,941 |
|  |  | *Anchoveta* respiration | 151,182,174 |
|  |  | *Anchoveta* mortalities | 21,185,411 |
|  |  | *Anchoveta* catches | 4,273,928 |
|  |  | *Anchoveta* un-assimilation | 97,394,503.44 |
| *Anchoveta* biomass | 13,928,640 | *Anchoveta* biomass | 13,928,640 |
| **Hake** |  |  |  |
| *Anchoveta* consumption | 2,397,941 | Hake consumption (predators) | 341,653.69 |
| Other prey consumption | 4,379,889 | Hake respiration | 1,948,507 |
|  |  | Hake un-assimilation | 2,054,765 |
|  |  | Hake mortalities | 575,177 |
|  |  | Hake catches | 99,843 |
| Hake biomass | 952,741 | Hake biomass | 952,741 |


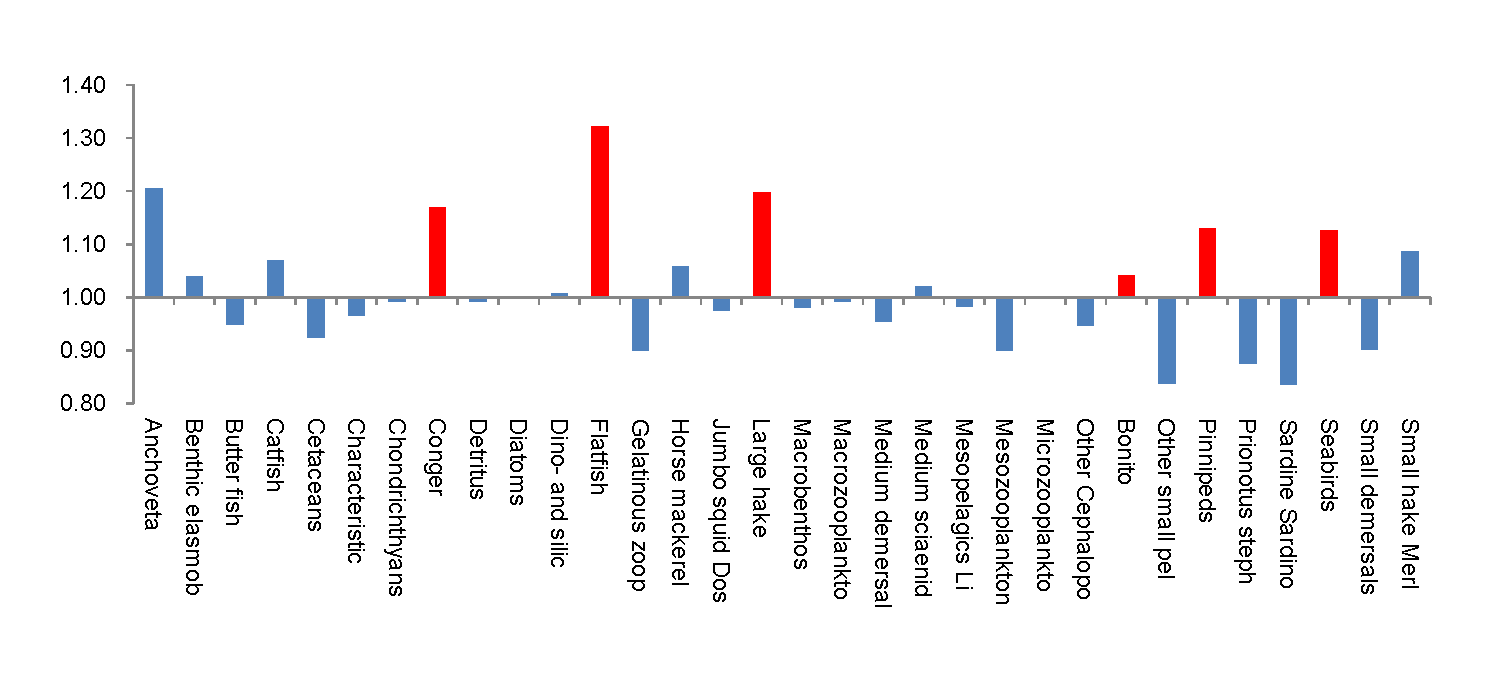


**Figure C1.** Changes in biomasses of all modelled species from 2004 to 2031, after a 50% reduction of *anchoveta* fishing mortality.

**
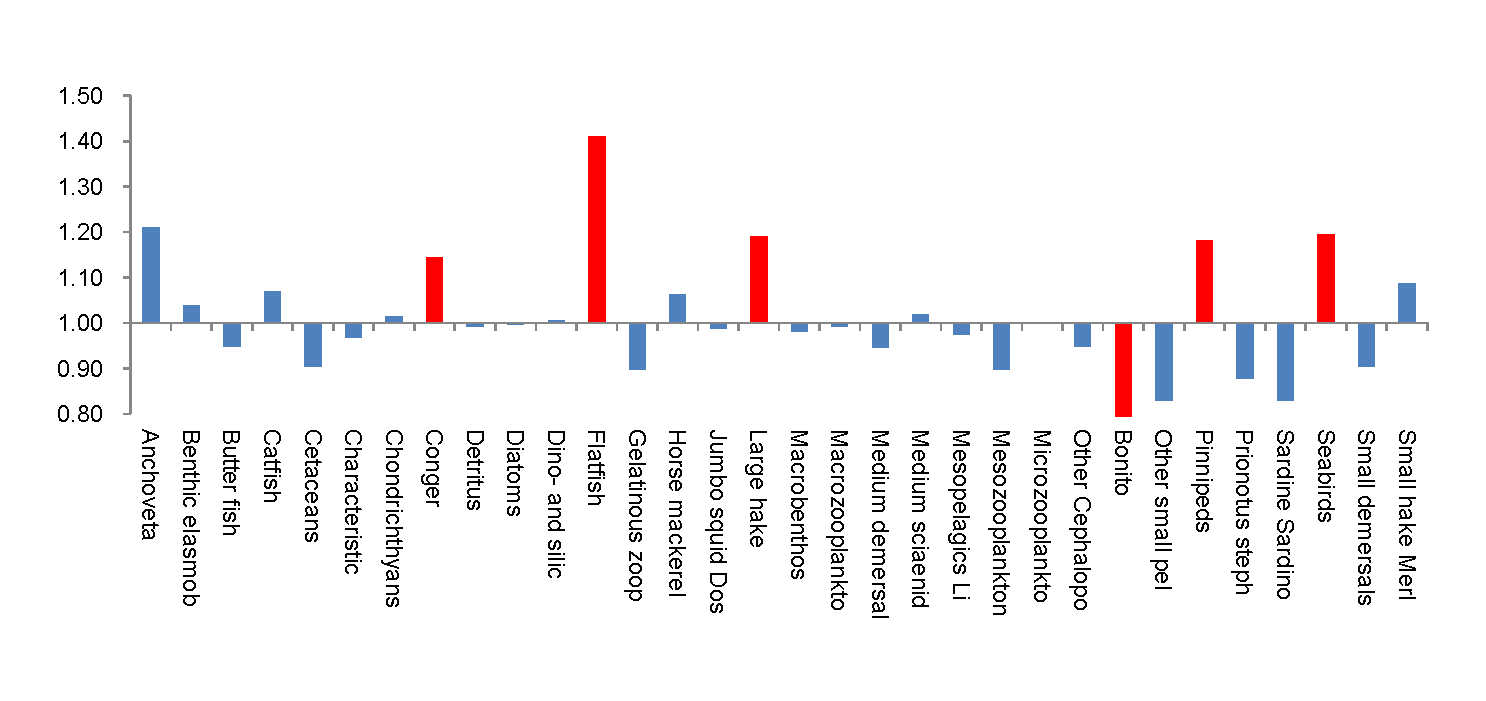
**

**Figure C2.** Changes in biomasses of all modelled species from 2004 to 2031, Scenario 3. Fishing mortality of hake increased in 22% and of bonito in 45%, proportional to the biomass increase in Figure C1.

**
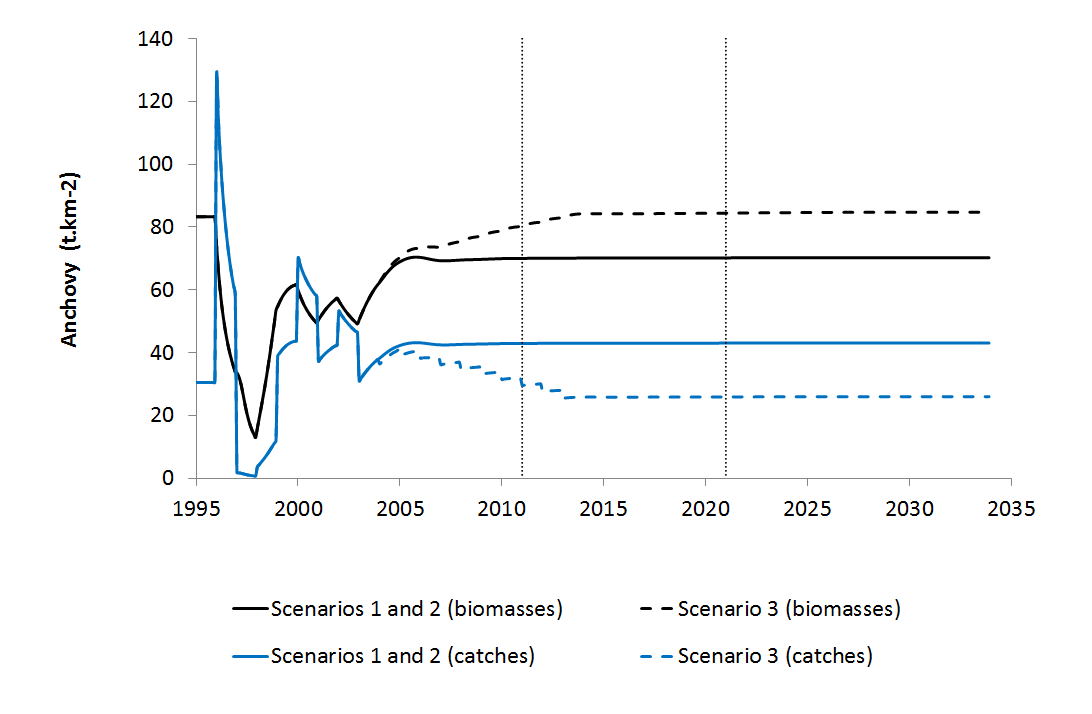
**

**Figure C3.** Comparison of *anchoveta* biomasses and catches among scenarios.

**
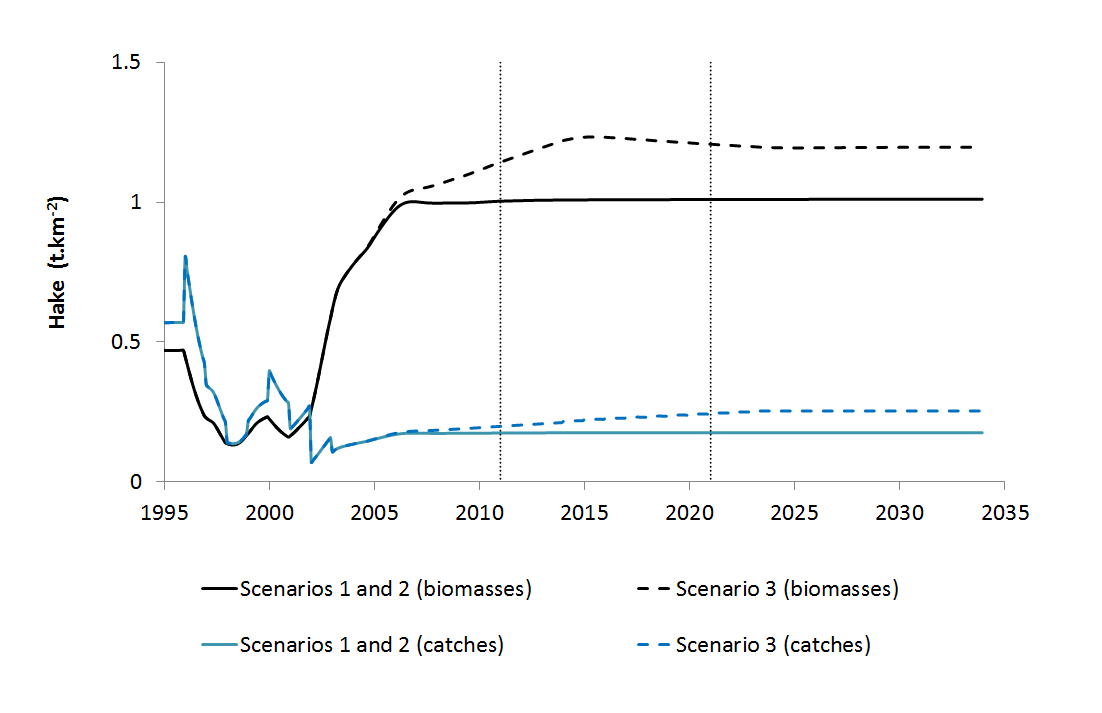
**

**Figure C4.** Comparison of hake biomasses and catches among scenarios.

## References

1. Horan RD, Fenichel EP, Drury KLS, Lodge DM (2011) Managing ecological thresholds in coupled environmental-human systems. Proc Natl Acad Sci U S A 108: 7333–7338. Available: http://www.pubmedcentral.nih.gov/articlerender.fcgi?artid=3088591&tool=pmcentrez&rendertype=abstract. Accessed 2 March 2012.

2. Schlüter M, Mcallister R, Arlinghaus R, Bunnefeld N, Eisenack K, et al. (2012) New horizons for managing the environment: A review of coupled social-ecological systems modeling. Nat Resour Model 25: 219–272. doi:10.1111/j.1939-7445.2011.00108.x.

3. Plagányi ÉE (2007) Models for an ecosystem approach to fisheries. Rome: Food & Agriculture Org. Available: ftp://ftp.fao.org/docrep/fao/010/a1149e/a1149e.pdf. Accessed 20 May 2011.

4. Shin Y-J, Cury P (2001) Exploring fish community dynamics through size-dependent trophic interactions using a spatialized individual-based model. Aquat Living Resour 14: 65–80. Available: http://linkinghub.elsevier.com/retrieve/pii/S0990744001011068. Accessed 26 May 2011.

5. Fulton E, Fuller M, Smith A (2004) Ecological indicators of the ecosystem effects of fishing: final report. Australian Fisheries Management Authority. Available: http://www.sainsolutions.net/Ecol Ind _simulation testing.pdf. Accessed 30 April 2012.

6. Rose KA, Allen JI, Artioli Y, Barange M, Blackford J, et al. (2010) End-To-End Models for the Analysis of Marine Ecosystems: Challenges, Issues, and Next Steps. Mar Coast Fish Dyn Manag Ecosyst Sci 2: 115–130. Available: http://afsjournals.org/doi/abs/10.1577/C09-059.1. Accessed 23 August 2010.

7. Allen JI, Fulton E (2010) Top-down, bottom-up or middle-out? Avoiding extraneous detail and over-generality in marine ecosystem models. Prog Oceanogr 84: 129–133. Available: http://linkinghub.elsevier.com/retrieve/pii/S0079661109001499. Accessed 7 July 2011.

8. Fulton E (2010) Approaches to end-to-end ecosystem models. J Mar Syst 81: 171–183. Available: http://linkinghub.elsevier.com/retrieve/pii/S0924796309003509. Accessed 20 May 2011.

9. Travers M, Shin Y-J, Jennings S, Machu E, Huggett J a., et al. (2009) Two-way coupling versus one-way forcing of plankton and fish models to predict ecosystem changes in the Benguela. Ecol Modell 220: 3089–3099. Available: http://linkinghub.elsevier.com/retrieve/pii/S0304380009005766. Accessed 18 May 2011.

10. Kleijnen J (2005) Supply chain simulation tools and techniques: a survey. Int J Simul Process 1: 82–89. Available: http://inderscience.metapress.com/index/NAR9RN3062RQ7A30.pdf. Accessed 26 April 2012.

11. Shapiro JF (2000) Supply chain management, integrated planning, and models. Modelling the supply chain. Cengage Learning. pp. 1–42.

12. Keramati AA (2010) Supply chain integration: A modelling classification. Eighth Annual International Symposium on Supply Chain Management. SCMP - Supply Chain Management Professional. Available: http://amir-keramati.com/so/SympoCanadfinal version.pdf. Accessed 30 April 2012.

13. Keramati AA, Eldabi T (2011) Supply Chain Integration : Modelling Approach. In: Ghoneim A, Themistocleous M, Koufopoulos D, Kamal M, editors. Online Proceedings of the 8th European, Mediterranean & Middle Eastern Conference on Information Systems (EMCIS). Athens, Vol. 2011. pp. 75–85. Available: http://www.iseing.org/emcis/EMCISWebsite/EMCIS2011 Proceedings/SCM8.pdf.

14. Beamon B (1998) Supply chain design and analysis: Models and methods. Int J Prod Econ 55: 281–294. Available: http://www.sciencedirect.com/science/article/pii/S0925527398000796. Accessed 30 April 2012.

15. Min H, Zhou G (2002) Supply chain modeling: past, present and future. Comput Ind Eng 43: 231–249. Available: http://linkinghub.elsevier.com/retrieve/pii/S0360835202000669.

16. Ahumada O, Villalobos JR (2009) Application of planning models in the agri-food supply chain: A review. Eur J Oper Res 196: 1–20. Available: http://linkinghub.elsevier.com/retrieve/pii/S0377221708001987. Accessed 29 October 2012.

17. Acar Y, Kadipasaoglu S, Schipperijn P (2010) A decision support framework for global supply chain modelling: an assessment of the impact of demand, supply and lead-time uncertainties on performance. Int J Prod Res 48: 3245–3268. doi:10.1080/00207540902791769.

18. Kasi V (2005) Systemic Assessment of SCOR for Modeling Supply Chains. Proceedings of the 38th Annual Hawaii International Conference on System Sciences. Ieee, Vol. 00. p. 87b–87b. Available: http://ieeexplore.ieee.org/lpdocs/epic03/wrapper.htm?arnumber=1385413.

19. Kim C, Tannock J, Byrne M, Farr R, Cao B, et al. (2004) Sate of the art review: Techniques to model supply chain in an extended enterprise.

20. SCC (2010) Supply Chain Operations Reference (SCOR ®) model - Overview - Version 10.0. Supply Chain Council.

21. Lavassani KM, Movahedi B (2010) Critical Analysis of the Supply Chain Management Theories: Toward the Stakeholder Theory. POMS 21st Annual Conference. Vancouver: Production and Operation Managemet Society. Available: http://www.pomsmeetings.org/ConfProceedings/015/FullPapers/015-0545.pdf.

22. Aramyan L (2007) Measuring Supply Chain Performance in the Agri-Food Sector Wageningen University. Available: http://library.wur.nl/WebQuery/edepot/121904.

23. Deep A, Dani S (2009) Managing Global Food Supply Chain Risks: A Scenario Planning Perspective. POMS 20th Annual Conference. Orlando: Production and Operation Management Society. pp. 1–21. Available: http://www.pomsmeetings.org/confproceedings/011/fullpapers/011-0371.pdf. Accessed 25 April 2012.

24. Van der Vorst JG a. J, Beulens A (1999) A Research Model for the Redesign of Food Supply Chains. lnternational J Logist Res Appl 2: 161–174. doi:10.1080/13675569908901579.

25. Dabbene F, Gay P, Sacco N (2008) Optimisation of fresh-food supply chains in uncertain environments, Part I: Background and methodology. Biosyst Eng 99: 348–359. Available: http://linkinghub.elsevier.com/retrieve/pii/S1537511007003510. Accessed 2 April 2012.

26. Jensen TK, Nielsen J, Larsen EP, Clausen J (2010) The Fish Industry—Toward Supply Chain Modeling. J Aquat Food Prod Technol 19: 214–226. Available: http://www.tandfonline.com/doi/abs/10.1080/10498850.2010.508964. Accessed 13 March 2012.

27. Parfitt J, Barthel M, Macnaughton S (2010) Food waste within food supply chains: quantification and potential for change to 2050. Philos Trans R Soc Lond B Biol Sci 365: 3065–3081. Available: http://www.pubmedcentral.nih.gov/articlerender.fcgi?artid=2935112&tool=pmcentrez&rendertype=abstract. Accessed 9 March 2012.

28. Bjørndal T, Lane DE, Weintraub A (2004) Operational research models and the management of fisheries and aquaculture: A review. Eur J Oper Res 156: 533–540. Available: http://linkinghub.elsevier.com/retrieve/pii/S0377221703001073. Accessed 26 April 2012.

29. Mai N, Bogason SG, Arason S, Árnason SV, Matthíasson TG (2010) Benefits of traceability in fish supply chains – case studies. Br Food J 112: 976–1002. Available: http://www.emeraldinsight.com/10.1108/00070701011074354. Accessed 14 March 2012.

30. Börjeson L, Höjer M, Dreborg K-H, Ekvall T, Finnveden G (2006) Scenario types and techniques: Towards a user’s guide. Futures 38: 723–739. Available: http://linkinghub.elsevier.com/retrieve/pii/S0016328705002132. Accessed 13 July 2012.

31. Arias M (2012) The evolution of legal instruments and the sustainability of the Peruvian anchovy fishery. Mar Policy 36: 78–89. doi:10.1016/j.marpol.2011.03.010.

32. De la Puente O, Sueiro JC, Heck C, Soldi G, de la Puente S (2011) La Pesquería Peruana de Anchoveta - Evaluación de los sistemas de gestión pesquera en el marco de la certificación a cargo del Marine Stewardship Council (The Peruvian anchoveta fishery - Assessment of the fishery management systems in the framework of th. Universidada Peruana Cayetano Heredia, Centro para la Sostenibilidad Ambiental. Available: http://www.csa-upch.org/pdf/lapesqueriaperuana.pdf.

33. PAD (2008) Realidad Pesquera de la Flota Industrial de Madera: Mitos y Verdades. Escuela de Dirección, Universidad de Piura.

34. Paredes CE, Letona Ú (2013) Contra la corriente: La anchoveta peruana y los retos para su sostenibilidad. Lima: World Wildlife Fund (WWF) and Universidad de San Martín de Porres (USMP). Available: http://awsassets.panda.org/downloads/anchoveta_version_final.pdf.
